# Helping or watching it happen: how participants respond to robot failures in a turn-taking game

**DOI:** 10.3389/frobt.2025.1664334

**Published:** 2026-02-06

**Authors:** Samantha Stedtler, Katherine Harrison, Valentina Fantasia

**Affiliations:** 1 Department of Philosophy and Cognitive Science, Lund University, Lund, Sweden; 2 Department of Thematic Studies (TEMA), Linkopings Universitet, Linköping, Sweden

**Keywords:** feminist STS, interactional repair, multimodal conversation analysis, robot failures, social robotics

## Abstract

Robot failures in Human-Robot Interaction (HRI), though often stemming from technical limitations, can have severe effects on the interactional dynamics between humans and robots. Prior empirical research has led to conflicting findings on how such failures influence user perceptions and the overall success of the interaction. In this study, we investigate how human participants respond to robot failures on a moment-to-moment basis, with a particular focus on how social roles, responsibilities, and agency are negotiated as these episodes unfold. We examine how responses and helping behaviors are instantiated, and which factors facilitate or hinder recovery strategies. We focus on kinematic failures, such as interruptions in motion, unsuccessful grasping, or dropping objects, that occurred during Tic-Tac-Toe games between human participants (n = 17) and the humanoid robot Epi. Our analysis combines multimodal conversation analysis (MCA) and thick description, drawing on our interdisciplinary backgrounds in cognitive science and feminist Science and Technology Studies (STS). We present selected interactional sequences that illustrate a range of participant responses, including physical repair and scaffolding, interpretive support, emotional care, sustained monitoring, and dynamic negotiation of agency. These observations demonstrate how humans co-construct interactional continuity and robot competence through distributed, multimodal, and affective forms of help. They also reveal how agency is dynamically reconfigured, and how roles and responsibilities are distributed across human and robotic actors. We show how the burden of repair often falls to the human participant and conclude by reflecting on the setting and methods used, specifically in regards to the role of the robot as a research tool.

## 1 Introduction

Errors and breakdowns are common in human-robot interaction (HRI), particularly in interactions with users unfamiliar with robotic systems (De Graaf et al., 2017; Salem et al., 2015; Lee and See, 2004). These disruptions can stem from slow processing, hardware malfunctions, software bugs, or human misinterpretation (Tian and Oviatt, 2021; Honig and Oron-Gilad, 2018; Žunjić, 2018). Interestingly, some studies suggest that robot errors can occasionally increase a robot’s relatability or likability, while for instance trust and task performance can be negatively affected (Harrison et al., 2025; Reig et al., 2021; Mirnig et al., 2017; Ragni et al., 2016). These contradictions highlight the need for a closer, moment-to-moment analysis of how people experience and respond to robot errors in practice. In real-world contexts, people often distribute the burden of compensating for such errors among themselves (Pelikan and Hofstetter, 2023). Many of these compensation strategies can be categorised as helping and repair behaviors; particularly repair is crucial for resolving communication breakdowns (Albert and De Ruiter, 2018; Denis et al., 2015). In human-robot interaction, however, the burden of repair often fully shifts to the human partner due to robots’ limited social, normative and agentic competencies (Pelikan et al., 2024; Mutlu and Forlizzi, 2008), highlighting the need to view repair as a relational and ethical practice shaped by broader sociotechnical dynamics (Arnelid, 2025; Lipp, 2024). In addition, this additional labor may lead to cognitive or physical efforts and cause frustration and fatigue (Mansoor, 2025; Zhou et al., 2017).

From a feminist Science and Technology Studies (STS) perspective, repair and maintenance are embodied, often invisible forms of labor disproportionately performed by marginalized groups (Graziano and Trogal, 2019; Graham and Thrift, 2007). The feminist STS lens foregrounds how ethics and power are organized in situated, triadic HRI, where protocols, scripts, and maintenance practices shape who may notice, name, and fix problems (Winkle et al., 2023; D’ignazio and Klein, 2023). Technologies are understood as designed and maintained within specific sociocultural contexts and thus as reflecting, and often reproducing, prevailing norms (Haraway, 1994; Harding, 2009; Barad, 2001). According to that perspective, seemingly autonomous systems rely on ongoing human care which reshapes the human-robot relationship and risks reinforcing existing power structures and social inequalities (Ostrowski et al., 2025; Schwennesen, 2019). One goal is therefore to make visible the care and repair work that typically recedes into the background, such as facilitators’ staging, researchers’ ratifications, and participants’ micro-help (Iversen et al., 2025; Pelikan et al., 2024; Chevallier, 2023; Blond, 2019). This study draws from this perspective to investigate how participants respond to kinematic errors during a turn-taking game with the humanoid robot Epi. Following (Tian and Oviatt, 2021) taxonomy of social errors, rather than treating errors as mere technical glitches, we conceptualize them as socially situated events, negotiated in real time as they emerge within human-robots interactions. At issue in this work is not only how helping behaviours are designed or displayed, but also which aspects may facilitate or hinder those, the roles humans and Epi assume, and the broader spatial and temporal organization of these interactions. Through a multimodal qualitative video analysis, we explore how participants use embodied and verbal resources and the simultaneous engagements of bodies and features of the material environment (Goodwin, 2018), to manage potential breakdowns, and how contextual factors shape their repair behavior. While quantitative approaches offer generalizable insights, they risk reducing the complexity of human behavior into standardized categories, which may miss the sequential and situated nature of help, repair, and interpretation (Veling and McGinn, 2021; Goodwin, 2018; Suchman, 2007; Verschuren, 2001; Winograd and Flores, 1986). By contrast, our analysis embraces variability and complexity, aiming to uncover the nuanced and embodied ways people make sense of, and respond to robot(ic) errors. Our two-step methodological approach combines thick description with multimodal conversation analysis (MCA), based on video-recorded sessions of participants playing Tic-Tac-Toe against Epi. Positioned at the intersection of cognitive science and feminist Science and Technology Studies (STS), this work also reflects on how these perspectives complement and challenge each other. This interdisciplinary lens not only enriches our understanding of spontaneous human behavior in HRI, but also offers implications for ethical considerations when preparing for robot failures.

## 2 Background

### 2.1 Failures in HRI

Understanding robot failures requires bridging system-level classifications with participants’ lived experiences. Failures in Human-Robot Interaction (HRI) are complex, multifaceted events that disrupt expected functionality and introduce uncertainty for interactants (Tabatabaei et al., 2025; Garza, 2018). From a more technical standpoint, errors are problematic system states that may lead to failures (Honig and Oron-Gilad, 2018). Here, we use the terms error and failure interchangeably, reflecting the frequent ambiguity in identifying the source of breakdowns. Honig and Oron-Gilad (2018) propose a useful distinction between technical failures, such as hardware malfunctions or software errors, and interaction failures, which emerge from ambiguities in the robot’s engagement with humans, other agents, or the environment. They also argue that any failure, regardless of its origin, can be characterized by attributes such as functional severity, social severity, relevance, frequency, and the conditions or symptoms under which it occurs (Honig and Oron-Gilad, 2018). Other scholars have similarly proposed classifications based on recoverability, severity, and failure type (Giuliani et al., 2015; Steinbauer, 2012; O’Hare et al., 2004; Laprie et al., 1995). While useful from a system design standpoint, such taxonomies are less accessible to end users, who must often infer causes and responsibilities based only on the observable behavior of the robot (Honig and Oron-Gilad, 2018; Kim and Hinds, 2006) as well as expectations and common narratives (Rhee, 2018). From interactants perspective, the boundaries between error and intentional behavior is often blurred, especially when robots are assigned social agency (Van Der Woerdt and Haselager, 2019). This ambiguity creates uncertainty, not just about what went wrong, but also about how to respond. Both experts and laypersons commonly struggle to link observable symptoms to underlying causes (Steinbauer, 2012). In some cases, technical failures on the robot’s part can even lead to (perceived) social norm violations by the human, particularly when implicit expectations arise about who should step in to resolve the problem (Stedtler and Leventi, 2025). How participants perceive failures also shapes their trust in the robot and their willingness to re-engage with it in the future (Ragni et al., 2016; Hamacher et al., 2016; Lee et al., 2010). Effective mitigation strategies (explanations or repair attempts) can help restore trust, while the absence of such responses may lead to breakdowns in interaction.

### 2.2 Helping and repair

In human interaction, repair and helping behaviors are essential for sustaining interaction and task flow (Albert and De Ruiter, 2018; Greiffenhagen and Watson, 2009). Repair refers to practices for resolving breakdowns in conversations, e.g., misunderstandings, mispronunciations, or action failures (Hayashi et al., 2013; Kitzinger, 2012), while helping encompasses broader forms of support shaped by moral, social, and psychological factors, as well as context and individual traits (Miller, 2010; Wagner and Wheeler, 1969). Helping behavior can signal and support in-group membership and social bonds (Lefevor and Fowers, 2016). This behavior may contribute and be determined by the emergence of social roles, which are patterns of behavior recognized by interaction partners, corresponding to shared expectations about their own and others’ behavior (Schindler, 2024; Vinciarelli et al., 2011). When it comes to roles for participants and robots in HRI, the following roles have been identified by previous researchers: peer, coperator, collaborator, learner, imitator, interviewee, mentor, supervisor, operator, mechanic, information consumer and bystander (participant) and peer, learner, tutor, mediator, assistant, interview, demonstratoor and testbed platform (robot) (Onnasch and Roesler, 2021; Goodrich and Schultz, 2008; Yanco and Drury, 2004). In addition to responding to normative expectations, helping and repair in human interaction can reflect more pragmatic concerns, such as energy preservation due to anticipated costs of unresolved problems (Anderson, 2003). During communication, repair mechanisms address issues in producing, perceiving, and interpreting turns (Dingemanse and Enfield, 2024). A common strategy is self-repair, but partners also engage in interactive repair, where one participant marks the problem and the other provides a solution (Albert and De Ruiter, 2018; Hayashi et al., 2013). These processes help secure mutual understanding and preserve the flow of interaction. In conversation analysis, accountability refers to making one’s actions intelligible and interpretable to others; when social norms are violated, this accountability becomes particularly salient (Dingemanse and Enfield, 2024). While repair plays a crucial role in maintaining interaction, it is also costly to the agent performing it. As a result, people sometimes avoid repairs or offer noncommittal responses to minimize disruptions, a phenomenon described as “organizational slack.” (Dingemanse and Enfield, 2024). Embodied repair work is often shaped by spatial configuration, proximity of the agents, and visibility of the error (Heath et al., 2010). In HRI, this spatial dimension can shape who feels responsible or able to intervene. Turn-taking games present an intricate and pre-defined structure for coordination that require participants to closely monitor the actions of others (Hoffman et al., 2014). Timing in natural interactions is usually well-maintained by the interaction partners; for instance, the concept of multimodal interaction management describes how participants work together to keep a conversation flowing smoothly and in sync (Brône et al., 2017). Central to this process is its sequential organization (Sacks et al., 1974). Sequence organization refers to the structured turn-taking that ideally produces alternating speaker turns with minimal overlap, which ensures clear and effective communication. At the same time, spatial organization of the interaction can equally determine how actions unfold and follow after each other. Goffman (2023)’s ‘territories of the self’, such as personal space, object positioning and the turn (a situational claim to act or speak), describe discrete social territories that, when disrupted (for instance, by a robot encroaching or interrupting), require repair to re-establish interactional order. Goodwin (2000)’s study of “Chil,” a stroke survivor with severely limited speech, shows how agency and repair can be distributed across bodies and interactional space. Even with minimal verbal resources, Chil was able to participate meaningfully in conversation and maintain agency by relying on the sequential structure of talk and the embodied support of others. In human-human interaction, repair is typically shared, emerging from mutual accountability and coordinated social stakes. Participants rely on predictable conversational structures to signal and resolve problems (Hollan et al., 2000). In human-robot interaction (HRI), however, this dynamic changes. Robots lack the normative grounding, social accountability, and flexible interpretive skills that support collaborative repair, and thus, the burden of repair frequently falls on the human partner (Pelikan and Hofstetter, 2023). These moments of failure, while asymmetrical, can become opportunities for relational engagement, helping to bridge the gap between robot imaginaries and the realities of current systems (Harrison et al., 2023). They also draw attention to the human labor that sustains technological functioning. Science and Technology Studies (STS), as a field, has for several decades explored precisely these dynamics. Whilst many scientific disciplines are concerned with the end result of an experiment or investigation, this scholarship is relevant here as it acknowledges the fragility of technical systems and the need for human repair work. While earlier STS work focused on the role of technicians and maintenance of technical or scientific equipment (Mukerji et al., 1991; Latour et al., 2013), later strands reflected on the value and visibility of such work, bringing an explicit attention to power structures (Star and Strauss, 1999; Shapin, 1989). More recently, STS scholars have connected repair practices to the burgeoning literature on care:

“Repair: The subtle acts of care by which order and meaning in complex sociotechnical systems are maintained and transformed, human value is preserved and extended, and the complicated works of fitting to the varied circumstances of organizations, systems and lives is accomplished” (Jackson, 2014, p. 222)

The excerpt above from Jackson notably elevates acts of repair to include ethical questions of human value and normativity (see also Graham and Thrift (2007), de La Bellacasa (2011)), and reframes repair work as part of a relational dialogue. It is this nuanced attention to failure in human-robot interaction and the ensuring repair work (or lack of it) (Harrison et al., 2025; Harrison and Johnson, 2023) that informs the interdisciplinary analysis in this paper.

### 2.3 Critical perspectives on repair in HRI

As scholars like Suchman (2007), de La Bellacasa (2011) and Turkle (2011) pointed out, technologies that promise remedies to human vulnerabilities are very enticing. Yet, seemingly flawless technologies depend on care and maintenance routines, typically upheld by invisible labor from women and marginalized groups, which becomes problematic for HRI since these interactions tend to mirror broader social inequalities (Ostrowski et al., 2025). This raises ethical concerns about assigning tasks to autonomous systems without recognizing the human labor that supports them (Stedtler, 2025; Spektor et al., 2021). Denis et al. (2015) and Denis (2019) point out that examining the distribution and nature of helping and repair labor is particularly important due to its highly embodied and situated character. For example, they describe how migrant waste pickers and ‘hackers’ in collective repair workshops engage in exploratory processes, progressively identifying material properties and artifact vulnerabilities through attentive, hands-on testing. These practices involve situated sensing and manipulation: material enactments that are inherently tied to the bodies of those performing them. Similarly, carers are often required to physically reenact such embodied labor repeatedly in their everyday work (Casilli, 2025; Denis et al., 2015). Recent work in HRI and STS has emphasized the importance of scaffolding and emotional labor in supporting successful robot deployment. For example, Thunberg (2024) describes how care workers in eldercare homes constructed narratives around robotic companions to encourage emotional connection and engagement from residents, which can be seen as scaffolding performed by human facilitators. Similarly, Harrison and Johnson (2023) draw attention to the socioeconomic and infrastructural privileges required to repair or adapt domestic robots, e.g., through reorganizing a living space or modifying outdoor environments to make it possible for the robots to fulfill their tasks. This shows how the success and adaptation of robotic technology depends not only on technical reliability but on a network of human labor, social positioning, and material affordances, which are often taken for granted or erased in dominant narratives of automation (Spektor et al., 2021). Moreover, the ability and willingness to assist a robot are not evenly distributed. Factors such as bodily ability, technical literacy, familiarity with robotic systems, and comfort within specific sociocultural settings all shape who feels comfortable, and with that, possibly confident enough to intervene (Halim et al., 2022; De Graaf et al., 2017; Rosenthal-von der Pütten et al., 2013; De Graaf and Allouch, 2013; Robertson, 2010; Nomura et al., 2008; Suchman, 2007).

### 2.4 The present study

As robot technologies are increasingly deployed in domains such as care, education, and therapy, contexts that often involve vulnerable individuals, failures in human-robot interaction carry not only functional but also ethical consequences (Harrison et al., 2025). Understanding how people help and repair in response to these failures is thus not only important for improving design, but also for examining who takes on the work of repair, under what conditions, and with what consequences. Robot failure becomes a diagnostic moment, revealing how agency, accountability, and labor are distributed within the interaction, and highlighting for whom the system is robust, or fragile, under strain. This includes both the emotional labor of maintaining social order and the practical labor of keeping the task on track. While designers categorize failures by technical parameters, users must interpret breakdowns in real time, often with limited cues. Our focus here is on this interpretive gap: how participants identify, respond to, and manage the ambiguity of errors during interaction. In a previously collected data corpus, video recordings were collected of participants playing Tic-Tac-Toe against a humanoid robot named Epi. Interest in the current research emerged from observations of a range of different participants’ reactions to robotic errors that were not captured in the behavioural coding scheme adopted in the previous study (anonymised for review). Given its exploratory nature, this study is intended to be hypothesis-generating rather than hypothesis-testing (Silverman, 1998). The analysis centers on the spatial and temporal organization of interaction, grounded in the following guiding questions:

- Q1: What forms does help take in response to robot failures, and how are these enacted over time within the interaction?
- Q2: What roles do participants take on during these interventions, and how does Epi’s role shift in the course of the interaction? How do agency and responsibility shift?
- Q3: What factors facilitate or hinder the situated emergence of processes of repair and helping by human participants?

## 3 Methodology

### 3.1 Study design and interaction setting

The study used data from an experiment conducted in a laboratory setting, where participants interacted with the humanoid robot Epi, developed by the Cognitive Robotics team (Johansson et al., 2020).

Epi has arms with five degrees of freedom each, and its fingers are angled so that they converge toward a single point (Johansson et al., 2020). This design allows the robot to grasp objects, an ability that was essential for our study, as Epi marked its spots in the game by placing balls on the grid. The experiment followed the Wizard-of-Oz paradigm, meaning that all of Epi’s movements were pre-recorded and selected in real time by the operator during the experiment (participants were only informed about it being remote-controlled after the interaction had finished). One of the researchers operated the robot and chose the movements, while following the principle of extending each match as long as possible (i.e., blocking the participant from winning while also leaving chances for winning). The other researcher present sat next to the operator and pushed out the next ball that Epi was meant to pick up from behind the curtain. That researcher was also responsible for adjusting the ball position if Epi did not manage to pick it up. When talking about “the researcher” (R) in our analysis, we only included the latter one, since only that person was visible in the video recordings. The interaction was video recorded from two different angles: one from the side of the table, and one from the front (facing the participant’s face). Seventeen individuals (11 women, 6 men), aged between 20 and 34 years (M = 26.2, SD = 4.5), took part in this exploratory study. They were recruited from a range of academic disciplines among students and staff at [anonymised for blind review] University. In terms of education, 2 participants had completed secondary school, 6 held a bachelor’s degree, and 9 had postgraduate qualifications. Regarding occupation, 6 were students, 2 were employed (full- or part-time), 2 were both employed and studying, and 3 were unemployed. All participants gave informed consent prior to the study and received compensation for their participation. The game Tic-Tac-Toe was chosen because of its simplicity, which allowed the study to focus on the effects of robot movement rather than complex game strategies. Its familiarity and social nature also supported the study’s aim of exploring Epi’s potential as a social agent. The setup positioned Epi on one side of a table, facing the participant. A simple 3 × 3 Tic-Tac-Toe grid was marked on the table. The game was played using colored balls, with the goal of forming a line of three balls either vertically, horizontally, or diagonally.

### 3.2 Procedure

**FIGURE 1:**
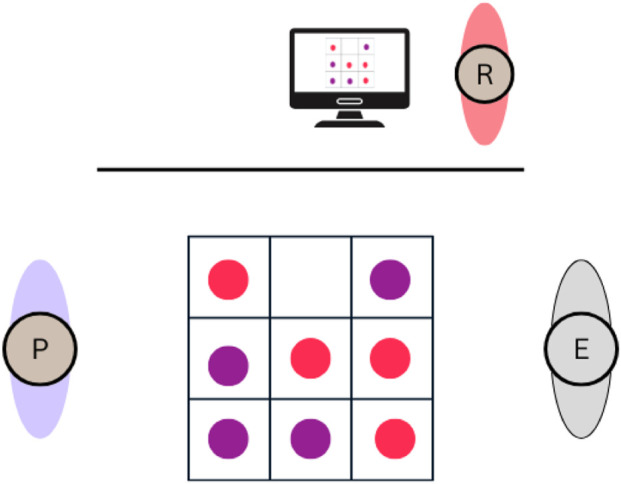
Task setting. P, participant; R, researcher; E, Epi. Reproduced from Stedtler et al. (2025), licensed under CC BY.

Upon arrival, participants were welcomed by the experimenter and asked to provide basic demographic information. They then completed questionnaires to assess their initial impressions of the robot’s social attributes. The participants played eight Tic-Tac-Toe matches against Epi. The game took place at a table, with a marked grid, where both Epi and the participant used balls to claim spots (see Figure 1). The goal was to form a row of three to win. For two-thirds of the participants, the robot displayed kinematic delays (4 s and 10 s) before placing the ball on the grid which were experimentally manipulated and occurred approximately during 30% of the game. The robot did not display any deliberate gaze behaviors, meaning it could not use eye gaze to signal its intentions or compensate for delays during the interaction. It only moved its head slightly in the direction it was turning, but these movements were largely a by-product of moving the rest of the body, without consistent or independent gaze control. This was done to keep the robot’s behavior as minimalistic as possible and avoid introducing confounding factors. As a result, the robot and participant only occasionally made eye contact, and such face-to-face moments were rare. Most of the time, the robot’s head was angled toward the board, giving the appearance of looking down. After the game, participants completed questionnaires regarding their perception of Epi’s social attributes again.

### 3.3 Approaches and frameworks

We used a qualitative approach, combining multimodal conversation analysis (MCA) and thick descriptions. MCA helped us examine how participants manage turn-taking and repair sequentially and spatially, while thick descriptions provided contextual depth by capturing the social and cultural meanings behind interactions. MCA seeks to understand how people produce and recognize meaningful actions in interaction, thereby establishing and maintaining social order through collaboration, incorporating gaze, gestures, material interactions, and spatial positioning (Heritage, 1984). From this perspective, communication is inherently situated and embodied, with meaning-making in on-going interaction being always co-constructed (Norris, 2004; Scollon and Scollon, 2003). Interaction management is a multimodal process (Brône et al., 2017). For instance, speakers use gaze to regulate who speaks when (De Ruiter, 2012; Kendon, 1967). Multimodality is closely linked to perception, yet traditional psychology has often studied senses in isolation (Jewitt, 2015). Howes (2019) critiques this approach, highlighting evidence from cognitive neuroscience and sensory anthropology that shows sensory modalities dynamically interrelated in perception. This simultaneousness of different modalities in interaction can be well transcribed and marked in the behaviour annotation software ELAN (Version 6.9; Max Planck Institute for Psycholinguistics, 2024). Disruptions offer a window into this process by making the normally invisible work of social organization visible, what Heritage (1984) calls “perspicuous settings”: These moments of troubles reveal how people use situated methods to manage and repair interaction, highlighting which norms were breached, why an action was treated as problematic, and how accountability is established locally (Rawls and Lynch, 2024). Our behavioural annotations in ELAN are accompanied by thick descriptions: originated in anthropology as a method for detailed ethnographic analysis, thick descriptions have since been adopted widely across qualitative research disciplines, including sociology, psychology, and education (Ponterotto, 2006). Thick description records not just what happens but also how experiences are shaped by history, emotion, and social relationships (Denzin, 1989). With that, it extends beyond reporting the basic facts of the robot’s body and actions towards the wider experimental space. It is a technique which produces a detailed account of a situation typically based on observation, but which extends beyond reporting the basic facts of what occurs in that space/moment. Norman Denzin, one of the key proponents of the term, describes thick description as follows:

A thick description … does more than record what a person is doing. It goes beyond mere fact and surface appearances. It presents detail, context, emotion, and the webs of social relationships that join persons to one another. Thick description evokes emotionality and self-feelings. It inserts history into experience. It establishes the significance of an experience, or the sequence of events, for the person or persons in question. In thick description, the voices, feelings, actions, and meanings of interacting individuals are heard. (Denzin, 1989, p. 83)

In qualitative research, then, this technique approaches any interaction as intimately connected with the surrounding context both spatially and temporally, and produces a record of the interaction which includes details of this. It acknowledges individual differences in how an environment is experienced due to surrounding sociocultural context. In the context of an HRI experiment, it shifts the primary focus from the body and actions of the robot to the wider experimental space in which human and robot are meeting. It takes into account that experiences with or representations of robots encountered prior to the experiment affect the human’s comfort interacting with the robot. Finally, it offers a challenge to the researcher to reflect on how their own presence and experience affects the interaction (Sergi and Hallin, 2011). Like other humanoid robots Epi is a work-in-progress designed and implemented to address research-led questions, and it is not uncommon for delays, glitches and failures in its functioning to occur. In this paper, we take these moments of malfunction and potential breakdown as analytical entry points. Emotions in interaction with AI and robotic technology have been foregrounded in recent critical feminist scholarship (e.g., D’ignazio and Klein, 2023; Persson et al., 2025). D’ignazio and Klein (2023) argue that critical engagement with the societal impact of technology requires elevating emotion and embodiment. They highlight how conventional approaches to data visualization often exclude precisely those aspects of human experience that are associated with emotion, affect, embodiment, expression, and even aesthetics. Because these dimensions have historically been linked with women, they have been devalued within dominant knowledge frameworks. A similar tendency can be observed in the empirical analysis of behavioral data: once complex, situated actions are translated into predefined categories and numbers, much of their richness and context is easily lost. If we instead treat participants’ actions as meaningful, their bodily postures appear to reflect different ways of engaging with Epi and to display varied affective expressions.

### 3.4 Data selection and mapping

The video data, collected by author 1 in a prior experiment, was reviewed to identify interactional patterns and episodes of interest, particularly those involving errors. Errors were defined as unplanned robot behaviors like dropping a ball. Author 1 compiled these segments into a catalog of key events [as described by Heath et al. (2010)], which were then shared with the two other authors. The segments chosen ended up being 26 in total, and lasting 15–30 s each. The segments analysed provided a view from the side (though sometimes they were complimented by snippets of the same event filmed from the front, e.g., to get a better view on the participant’s face). For a preliminary categorization, the segments were divided by helping (further categorized as moments of confusion, unnecessary helping and moments of reaching in the robot’s ‘territory’) vs. non-helping (in the end vs. not in the end of the match) behaviors. Also list other categories used for thick description and a note explaining why we chose these. All three authors collaboratively reviewed and refined this collection through data sessions and screenings, producing rough transcriptions and organizing the material around themes of helping and repair. The collection-building process was informed both by our research interests and by the data itself, for example, identifying certain practices and examining the routines in which they appear.

### 3.5 Transcription and data analysis

Within the selected data, detailed analyses were conducted using sequential multimodal transcriptions (i.e., multimodal interaction analysis) in ELAN (by author 1) and thick description (by author 2), with all three authors meeting regularly to compare insights across methods. This collaborative approach highlighted how different analytical lenses bring forward distinct aspects of behavior. The analysis focused on how help was offered, requested, accepted, or rejected, and how agency and normative roles were constructed in decision-making processes. The analysis consisted of an iterative process, with RQs being developed by all three authors during analysis and transcription as evolving flexible objects.

## 4 Error types and situations

Building on Honig and Oron-Gilad (2018) taxonomy of robot failures, Tian and Oviatt (2021) have developed a taxonomy of social errors in Human-Robot Interaction (HRI). This framework is particularly applicable to our scenario, as participants are not aware of the technical causes behind interaction problems but instead rely on their lived expertise with social scripts and competences required for successful interaction. According to Tian and Oviatt (2021)’s taxonomy, social errors include breaches in empathic and emotional reactions, insufficient social skills, misunderstandings of the user, insufficient communicative functions, and breaches in collaboration and prosociality. In our study, we distinguish between experimentally manipulated delays and spontaneous failures. The delays were systematically introduced as part of the experimental design, while the other failure types, such as mis-grasps or dropped objects, occurred unplanned and spontaneously across participants. These spontaneous errors were fairly evenly distributed among participants. Almost all situations described in this article involve these spontaneous failures. While previous empirical work (cited in the manuscript) focused on the experimentally induced delays, the current study centers on the naturally occurring, incidental errors and participants’ responses to them. In the delay section, such errors occurred in approximately 30% of trials, whereas spontaneous failures occurred at varying, incidental frequencies across the dataset. We observed several types of unplanned failures, including technical errors, which were always accompanied by socially-relevant breakdowns such as unresponsiveness, turn-taking disruptions, or incoherence, meaning that they fit well into this taxonomy. It also shows how technical failures in social contexts carry social consequences, especially when the robot fails to meet communicative or relational expectations. From a more technical point of view, the sequences we analysed contained the following robotic errors.

### 4.1 Delays

This was a variable which was experimentally manipulated to test participant reactions to different lengths of delays in the robotic movement for another research purpose. In the delay conditions, delays occurred 30% of the time. Some delays lasted 4 s and some 10 s. The latter was assumed to be more likely perceived as an error due to it being such a long waiting time. During the delay, the robot’s hand hovered above the grid before placing it, which could be interpreted as a thinking-pause as well. Sometimes the robot dropped the ball during a delay, making it more obvious that the delay is not a thinking pause (since that pause would be interrupted if the robot would ‘notice’ that it lost the ball).

### 4.2 Failing to pick up ball

**FIGURE 2:**
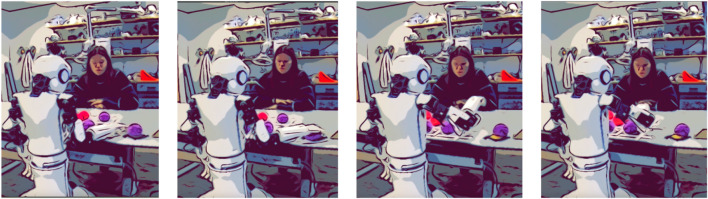
Epi failing at picking up a ball and moving its empty hand to the grid.

Since Epi (as of yet) does not have any sensors on its hands, the researchers had to physically, manually align the table with the ball-starting position with Epi’s body so that the hand movement would end up in the right place to pick up the ball. Due to various reasons, this sometimes failed, leading to Epi not being able to pick up the ball (see Figure 2). In those situations, Epi would still lead its hand towards the starting position of the ball, touching the ball while closing the hand, however not succeeding to capture to ball in its hand; thus, it performed the movement sequence without the ball: moving the empty hand to the grid, and opening it for the (non-existent) ball to drop in the right field. Sometimes, the ball would be dragged along for the first few millimeters, but outside of the hand.

### 4.3 Dropping ball in the middle of movement

This was often caused by, as described above, Epi not being able to fully grab the ball. Often Epi would succeed to have the ball somewhat in its hand, but then lose the ball on the way.

## 5 Responses to robot failures

Following Epi’s mistakes, we observed moments of interrupted helping and repair, often accompanied by negotiations around agency and the redistribution of responsibilities between Epi and the participant. These sequences involved subtle bodily coordination and shifts in affect, indicating a developing interactional stance.

### 5.1 Triangular interactions between participant, robot, and researcher

**FIGURE 3:**
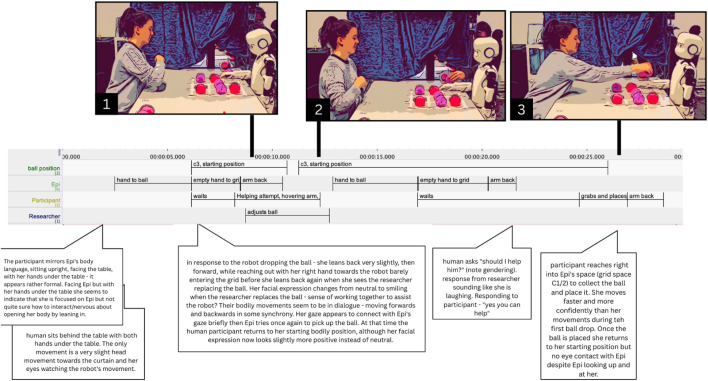
Participant P2 reacting to Epi failing to pick up the ball. Combined screenshots, transcriptions in ELAN and thick descriptions (in speech bubbles).

At the start, Participant P2 sits behind the table with both hands under the tabletop (see Figure 3). At 06.140 s, Epi fails to pick up the ball but continues its pre-programmed movement, extending its empty hand toward the grid (06.140–08.470 s). As Epi’s hand approaches the chosen spot, P2 leans slightly back (07.660 s), then forward (08.700 s), tentatively reaching out with her right hand (08.280 s), her fingers just barely entering the space, before pausing (09.910 s). Simultaneously, the researcher’s (R) hand emerges and adjusts the ball (08.710 s). P2 hovers her hand midair, watching this intervention (09.670–10.370 s). Her facial expression shifts from neutral to smiling (10.380 s), perhaps signaling shared recognition. The bodily movements of P2 and R appear loosely synchronized, both leaning forward and back in an emergent rhythm. This suggests that the responsibility for fixing Epi’s mistake is negotiated between P2 and R: P2 halts and interrupts her attempt as R intervenes. Epi tries to lift the ball again and fails (17.080 s). This time, P2 does not move during Epi’s action.

She waits (21.630–22.760 s) and finally asks: “should I help him?” (22.870 s). While her gaze remains on Epi, the question is addressed to the researcher. This reflects a shift in recipiency, creating a triangular interaction where P2 communicates with the researcher while focusing on Epi. The participant may no longer regard Epi as a full agent, and instead turns to the researcher. Alternatively, this behavior may respond to the researcher’s earlier intervention, which may have prompted the participant that the researcher’s involvement is appropriate and necessary.

**FIGURE 4:**
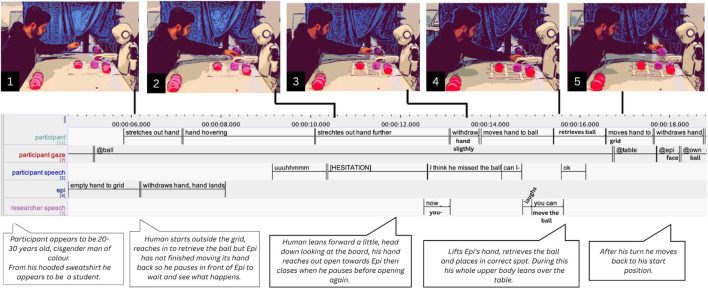
Participant P5 helping Epi placing its ball. Combined screenshots, transcriptions in ELAN and thick descriptions.

Yet, there were other instances of triangular communications that appeared without the researcher having interfered in any previous sequence. In one such incident, Epi loses the ball, moves its empty hand to the grid, and, after withdrawing, rests its hand on the lost ball. Participant P5 initially reaches out (5.860 s), getting very close to Epi’s hand, then hesitates and hovers over table (07.230 s), slightly withdrawing (09.410 s, see Figure 4). He utters “uhmm” (09.150 s), leading to R responding with “now you–,” (12.540 s) which is interrupted by P5’s “i think he missed the ball. Can I–?” (12.630 s), to which R laughs and replies, “you can move the ball” (14.940 s). P5 then retrieves the ball from under Epi’s hand (15.390 s) and places it on the intended spot (17.570 s). Although P5 reacts quickly, even before Epi has fully withdrawn, his hovering hand and verbal hesitation suggest uncertainty about the appropriate course of action. At the same time, he moves closer to Epi’s hand even before speaking, possibly to signal willingness to intervene.

### 5.2 Figuring out relevant norms

Several times, people appeared to signal both cooperation and uncertainty in regards to what norms apply to the situation. In the previously described (Section 5.1.1) fragments, this can be seen in participant P5 hesitating (Figure 4), as well as in P2’s request for clarification, but also in the way her question is phrased: “should I help him?” (see Figure 3), is a question one would not typically ask when for instance assisting a child; rather, it seems like a way of asking whether she is expected to help. The participant seems to be trying to figure out what obligations she has in the situation, or perhaps she is unsure whether intervening might disrupt the experimental protocol. Her arm hovering underlines this uncertainty as well. The researcher’s labor, in turn, involves clarifying and explaining how to act, i.e., making sense of the situation. Additionally, it seems this participant, too, is hesitant to touch or move Epi’s hand, which is a kind of work that we describe in the following sections (Sections 5.4, 5.5).

### 5.3 Treating robot actions as meaningful

**FIGURE 5:**
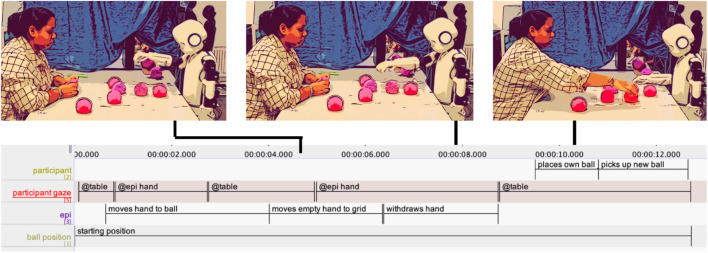
Participant P12 reacting to Epi failing to pick up the ball. Combined screenshots an transcriptions in ELAN.

We observed instances where Epi’s incomplete actions were interpreted as meaningful and consequential by participants. In one case, Epi fails to pick up the ball and performs the reaching motion toward a target grid space with an empty hand (04.140 s-06.360 s). Although the placement is not completed, Participant P12 appears to treat Epi’s movement as a legitimate turn: once Epi returns to its starting position (08.530 s), P12 waits briefly, then quickly places her own ball (09.520 s), not in the spot closest to her, but in the one furthest away, strategically blocking Epi from winning, had its previous move succeeded (see Figure 5). This suggests she takes Epi’s intentions seriously, treating them as completed actions that do not need correction. P12 is seated on the far left of the shared space with her forearms resting on the table. She has her final ball between her hands and passes it slowly between her fingers while waiting. Her posture is relaxed, slightly slouched forward, and her gaze alternates between Epi’s face and the board. Her expression remains neutral, with no signs of nervousness.

### 5.4 Adapting to timing and spatial relations

**FIGURE 6:**
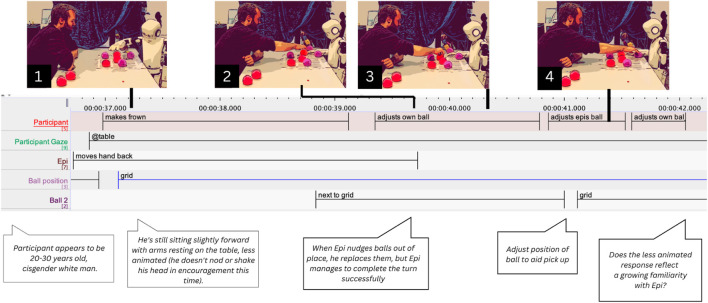
Participant P16 adjusting the balls after Epi nudged them away from their original place. Combined screenshots, transcriptions in ELAN and thick descriptions.

We also found that participants often perform actions that, from a purely gameplay perspective, are unnecessary. These behaviors contribute to maintaining order within the shared interactional space, such as adjusting balls that are not perfectly centered. One case involves Participant 16 (P16). During one of Epi’s turns, the robot successfully places its ball but, while withdrawing its hand, nudges two balls from earlier turns slightly outside the grid. While Epi’s hand is still in motion, P16 begins adjusting (39.390 s) his own displaced ball (see Figure 6). Once Epi’s hand stops (39.760 s), he adjusts the ball placed by Epi (40.810 s) and returns to make a final, subtle correction to his own ball (41.660 s). Compared to earlier rounds, P16 appears less animated, no longer nodding or shaking his head in encouragement. This more subdued demeanor may indicate growing familiarity with Epi, reflecting an adjustment in affect and bodily responsiveness that suggests acclimatization to the robot’s behavior and limitations. These subtle behaviors reveal a general form of engagement and co-maintenance of the interaction, where the participant performs small repair-actions that are oriented along the temporal and spatial trajectories of Epi’s hand movements.

**FIGURE 7:**
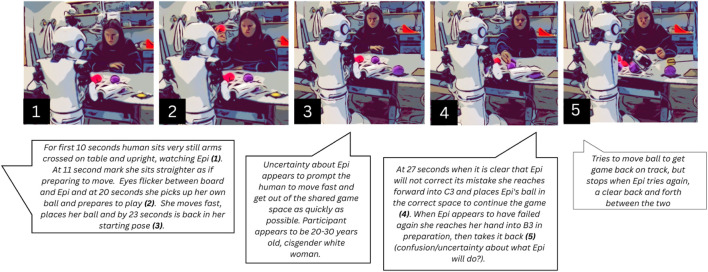
Participant P8 reacting to Epi failing to pick up the ball. Combined screenshots, transcriptions in ELAN and thick descriptions.

A similar situation occurs when in interaction with another participant (P8), Epi places a ball to the field closest to the starting position, slightly outside of the grid (06.920 s) and returns to its starting position, leaving its hand resting on top of the ball (09.600 s) (see Figure 7). P8 does not intervene immediately. She waits until Epi moves its hand again to pick up a new ball (26.310 s), and only then reaches forward to nudge the misplaced ball toward the center of the intended spot (27.230 s), inside the grid. When Epi again fails to pick up the ball in its next move (30.020 s), she prepares to intervene (32.040–33.790 s). A brief back-and-forth of interrupted repair attempts follows: she moves to help (38.530 s), stops as Epi tries again (39.780 s), and resumes her position (40.500 s). Once Epi succeeds, P8 immediately places her final ball (46.960 s). Just like P16, she engages in subtle moment-by-moment negotiations of repair that respond to the dynamics of Epi’s movements. Additionally, this scene reflects a pattern seen in other participants: avoiding placing their hand in the shared space while Epi’s hand is still present.

**FIGURE 8:**
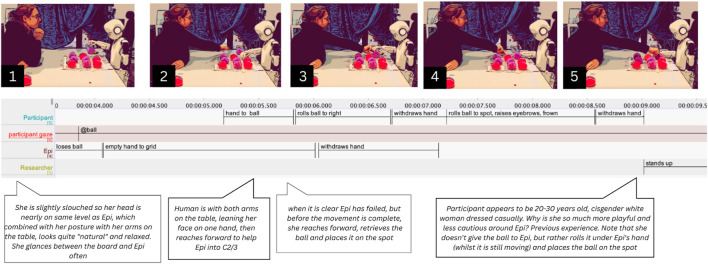
Participant P9 moving Epi’s ball to the grid.

In this example, P8 stops when Epi makes its moves, and proceeds to adjust misplaced balls when Epi has finished while carefully keeping a certain distance to Epi’s hands. However, there are some examples of participants moving simultaneously with Epi and in close proximity to its hand. For instance, during one turn, Epi drops the ball (03.640 s) but continues reaching (04.150 s) toward the grid (see Figure 8). P9 reaches toward the dropped ball (05.230 s), not to hand it to Epi or place it directly, but to gently roll it under Epi’s still-moving hand (05.840 s) and toward the intended spot (06.720 s). She times this carefully, avoiding interruption. When Epi’s hand nears hers (06.520 s), she briefly pulls back to avoid collision (06.730 s), then resumes nudging the ball (07.210 s). Her facial expressions, raised eyebrows, a brief frown (07.420 s), a smile (08.82 s), convey awareness of the awkwardness of intervening. Yet she appears less hesitant than others; rather than keeping her distance, she leans in and remains physically close to Epi. She treats the robot as a legitimate partner, hesitating to override it but stepping in when needed.

### 5.5 Help despite discomfort

**FIGURE 9:**
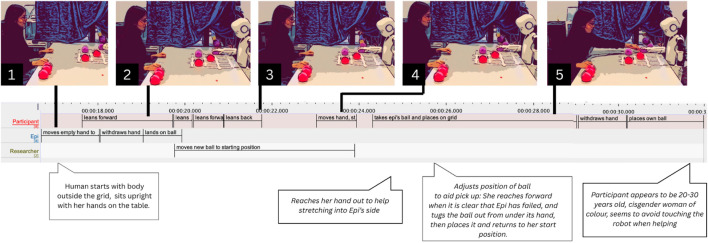
Participant P14 retrieving ball from under Epi’s hand and placing it.

In some cases, participants offer help to recover from failure, even when it involves physical discomfort or uncertainty. For instance, while participant P14 begins the interaction seeminlgy relaxed (seated upright outside the grid, hands resting on the table (see Figure 9)), as the game progresses, her body language reveals subtle signs of discomfort and negotiation around physical proximity to the robot.

When Epi fails to pick up the ball (16.130 s) and continues moving its empty hand toward the grid (16.790 s), P14 leans forward slightly (17.630 s) but quickly leans back as Epi’s hand lands on the ball close to its starting position (19.060 s). As the researcher adjusts the dropped ball behind the curtain, P14 leans forward again (20.210 s) but pulls back once more (20.890 s). These repeated forward and backward movements suggest an internal conflict between the felt obligation to help and a reluctance to encroach on Epi’s space. Eventually, she carefully reaches into Epi’s side of the grid (24.650 s), stretching her arm and fingers to tug the ball from under Epi’s hand (25.790 s) without touching the robot. She adjusts the ball’s position more centrally on the grid (28.370 s) and quickly returns to her original posture (29.130 s). Her movements highlight the social balancing act many participants engage in: offering assistance while considering their own comfort and boundaries. At times, participants help Epi even after the game outcome is decided, suggesting their actions go beyond ensuring the game to continue and instead are directed at enabling Epi to be successful in the sub-task of placing the ball.

### 5.6 Signaling readiness and empathy

**FIGURE 10:**
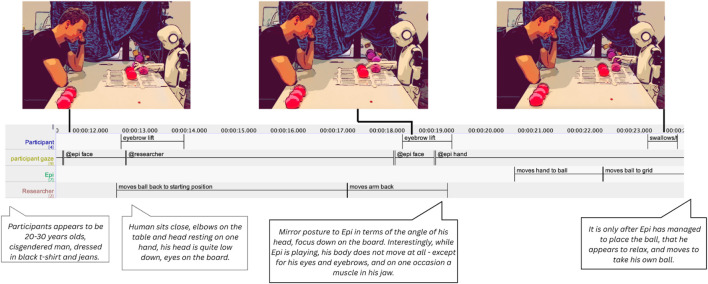
Participant P6 watching Epi struggle to place its ball.

Participants also responded to failures by staying alert and ready to intervene if needed. In one example, participant P6 does not physically assist Epi when it fails, but instead expresses concern through subtle bodily cues: he sits very still, raises and lowers her eyebrows at critical moments, tenses his face (visible in the movement of her jaw muscle), and maintains a heightened state of alertness (see Figure 10).

These embodied displays communicate availability, alignment, and effort, contributing to the social intelligibility of the interaction. The participant’s body functions not only as a sensor, registering moments of breakdown, but also as a resource for managing and anticipating errors: gaze direction, physical positioning, and readiness to touch all help scaffold the interaction and hold it together (Luff et al., 2000).

**FIGURE 11:**
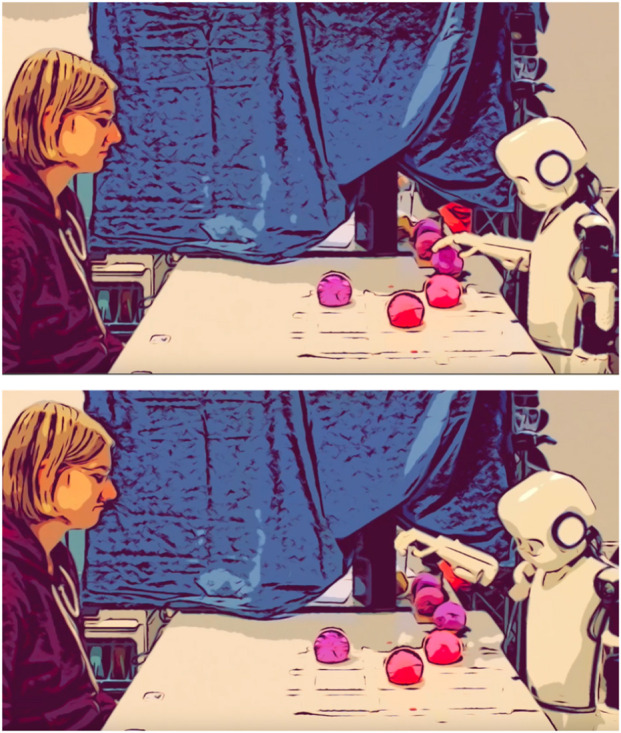
Looking sad at the robot if it does not succeed to pick up ball (P9).

**FIGURE 12:**
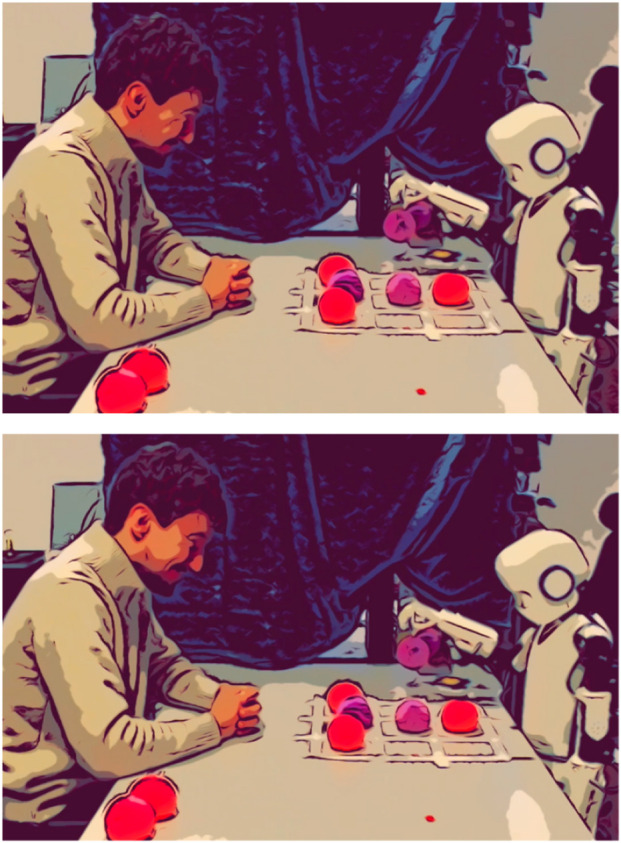
Smiling/laughing awkwardly (P15).

In addition, we observed affective displays performed by the participants during the interaction, as well as signs of emotion regulation. One kind of those was showing empathy through for instance displaying a sad expression at the robot if it does not succeed to pick up the ball (P9) (see Figure 11). Another kind of emotional displays seemed to rather deal with compensating for potential negative emotions and implications of the robot’s failed actions by smiling or laughing awkwardly (P15) (see Figure 12).

## 6 Discussion

### 6.1 What forms does help take in response to robot failures, and how are these enacted over time within the interaction? (Q1)

#### 6.1.1 Physical help and scaffolding

As Pelikan and Hofstetter (2023) describe, participants often engaged in subtle scaffolding, offering help and care without overtly taking over the robot’s role (see Table 1). These supportive actions maintained the illusion of Epi’s agency while ensuring the interaction continued smoothly (Spektor et al., 2021).

**TABLE 1:** Types of participant responses to robot errors and delays.

Response	Description
Physical help and scaffolding	Participants physically supported Epi by adjusting or replacing misplaced balls, sometimes anticipating future problems to preserve interaction flow
Interpretation and adaptation	Participants infered the robot’s intended actions and adjust their own behavior accordingly
Emotional and affective help	Participants displayed empathy, eased awkwardness, and offered gentle, caring help beyond task requirements
Maintaining the robot’s participation	Participants shifted between interacting with the robot and researcher, granting or withholding robot agency and taking or delegating responsibility depending on performance

Participants helped in response to robot failures by physically adjusting or replacing balls that Epi failed to place correctly or that were nudged out of place. Some adjusted balls after Epi placed or nudged them off-center (P8, P16), while others gently pushed a ball near Epi’s hand, showing careful scaffolding (P9). Sometimes help occurred even when not strictly necessary for the game-play itself, such as correcting a ball after the game ended or when placement was close enough (P14). At times, help was offered simultaneously, for example, the researcher stepping in at the same time as the participant, which created a conflict for the participant that had to be dealt with. In some cases, help was anticipated, like P16 (see Figure 6) fixing several balls nudged out of place to prevent bigger problems later. These acts can also be seen as a preventive and anticipatory form of help, preparing participants to step in as needed. This aligns with Anderson (2003)’s notion that such vigilance may serve to reduce the future cost of repair. P16’s re-adjustments, emotional responses, and micro-adjustments to prepare the environment for Epi’s next move can be seen as strategies to maintain interaction flow despite minor social misalignments, like failures in turn-taking or robot unresponsiveness. This also fits well with Denis et al. (2015)’s concept of maintenance by keeping order. Many physical repairs also occurred while Epi was still moving (e.g., P5), sometimes interrupted by unexpected events like researcher intervention. This repair often affirmed Epi’s agency by waiting for the robot to relinquish control before intervening, though not all participants waited.

#### 6.1.2 Interpretation and adaptation

As Dingemanse and Enfield (2024) emphasize, making one’s actions intelligible and accountable is crucial in social interaction. In Epi’s case, although its mistakes stem from technical issues, they result in social norm violations due to a lack of accountability. Consequently, participants must interpret and make sense of Epi’s actions themselves. Often, participants inferred Epi’s intended goals and acted based on those intentions even when the robot physically failed, for example, avoiding placing a ball in a spot Epi appeared to claim or adjusting behavior based on perceived intentions rather than outcomes (P8). Participant P12 similarly respected the intended placement despite Epi’s physical failure. As described in 5.1.2, there is a situation where Epi fails and instead of helping or stoping the interaction, P12 keeps playing as if Epi had succeeded with its move (the intended placement was indicacted by Epi’s empty hand being moved to the spot on the grid), in this case, by blocking Epi from winning. She does not offer help, yet still takes the robot’s intended action into account. This exemplifies what Denis et al. (2015) describe as moments of innovation tied to repair, where interactants invent ad-hoc rules to establish temporary order. This fits Dingemanse and Enfield (2024)’s concept of “organizational slack,” where individuals avoid repairs or offer noncommittal responses to minimize disruptions. P12’s behavior may reflect a choice to acknowledge Epi’s intention without interfering, preserving the robot’s agency and maintaining interaction flow. This reflects a cognitive and social form of help, where humans treat the robot as an intentional agent, adjusting their actions accordingly. Responsibility is actively assumed; participants do not wait for explicit instruction or prematurely intervene but contribute with awareness of Epi’s role. This distinction between responsibility through interpretation versus delegation is exemplified by P8, who directly interprets Epi’s intentions rather than deferring to others. Such behavior reinforces the robot’s role as participant and upholds a collaborative social script by compensating for its limited social skill, they thus engage in the “complicated works of fitting to varied circumstances” (Jackson, 2014, p. 222). This shows, recalling Goodwin (2000)’s study of Chil, how agency and repair are distributed across bodies and interactional space.

#### 6.1.3 Emotional labor, affective help and care

As Thunberg (2024) and Persson et al. (2025) have noted in their respective studies, successful human-robot interaction often involves an element of scaffolding or additional care that requires human (emotional) labor. We saw this manifested in our study in the form of participants seeming to show empathy, regulate their emotions, and perform actions of care for Epi. One could speculate whether the robot is truly the addressee of that care, or whether participants are using the robot to address a non-ratified participant (the researcher), who is not always officially called into the interaction. It may also be that displaying empathy and care is so socially ingrained that it is difficult to “switch off,” even when it seems irrational. This connects back to the concept of situated knowledge (Haraway, 2013): while robots in general might be described as neutral technological artifacts, the specific setting here, with Epi presented as child-like, clumsy, and noisy, encourages participants to act in ways that feel appropriate to this context. While such behavior is one way of familiarizing oneself with and relating to the technology, it can also become exhausting for the person performing this emotional work (Martínez-Iñigo et al., 2007). There were several instances where participants appeared to regulate the emotional climate of the interaction through displays of empathy or by managing the awkwardness caused by robot errors, for example, showing sadness or disappointment when Epi fails, or smiling and laughing awkwardly to ease tension or embarrassment. This constitutes a form of emotional labor that maintains the social bond and interactional stability despite failures, which is critical for sustaining collaboration. In terms of actions that could be seen as care or normative help, participants offered help that signaled care without overt task necessity, e.g., helping gently to encourage Epi rather than taking over (P9), or engaging in uncomfortable helping that balances social norms against personal discomfort (P14). For example, participants displayed conflicted body language when helping physically (P14). This resonates with Denis (2019), who describe how repair and maintenance work is embodied and situated. This work can be, and is, enacted with and on the worker’s body, or in this case, the participant’s body, even when it makes them potentially uncomfortable. At the same time, this embodied work helps participants figure out potential material vulnerabilities. This is also relevant here, as it is unclear how ‘fragile’ Epi’s body is, and the participants’ hesitancy may stem from not wanting to hurt or break Epi. In some instances, participants expressed concern non-verbally and tracked the robot’s progress with emotional involvement (P6, P9). This reinforces Epi’s role as a partner worth emotionally attending to.

Scholarship from within feminist STS provides a critical lens for examining such modes of caring for the robot, which bring attention to, for example, power imbalances. This is particularly pertinent when considering the emotional labour. For instance, Persson et al. (2025) have shown that caregivers perform emotional labor to facilitate interaction between care robots and dementia patients. Emotional labor constitutes a central dimension of care, insofar as it demands a readiness to be present and emotionally available for others. This readiness was observed in our participants as well, despite the robot’s inability to have feelings. With that, it also highlights reproduction of existing gender and other norms around care work: Acts of repair include ethical questions about human value and normativity (see de La Bellacasa, 2011; Graham and Thrift, 2007), and reframe repair work as part of a relational dialogue. Finally, we might also consider that the differences between the participants responses reflect their own sociocultural positions in terms of how and who can perform care.

#### 6.1.4 Maintaining the robot’s participation

Suchman (2008) points out that building truly embodied robots is hard to achieve since their functioning always depends on carefully constructed environments that predefine what they can perceive and how they can act. This leads to a conflict: while robots are often imagined and described as autonomous, their apparent agency in practice relies heavily on external scaffolding and human support (Spektor et al., 2021; Alač et al., 2011). In other words, the very conditions that make their actions possible also reveal their dependence on others to maintain their participation in the interaction. As mentioned earlier, Epi’s failures often evoked moments of negotiation and redistribution, both of Epi’s agency and of who is responsible for compensating for its mistakes. Epi’s agency could be extended by fulfilling actions in line with its perceived intentions, or withdrawn as the researcher stepped in to help. This negotiation unfolds sequentially, as participants’ perceptions of Epi’s agency shift over time, influenced by its performance and researcher interventions. Several clips show how help involves shifts in perceived agency. Sometimes participants bypass Epi by directing requests or gaze toward the researcher (P2), while at other times they engage directly with the robot, including in triangular interactions where the participant speaks to the researcher instead of the robot (P2). Similar triangulated interactions, where a caregiver looks at a child while speaking to a third party, have been documented in developmental research (Rogoff, 2003; Schieffelin and Ochs, 1986). Such dynamics illustrate a division of recipient roles, revealing how agency and responsivity are mediated (Goodwin, 2018; Tomasello and Carpenter, 2007). As Pelikan and Hofstetter (2023) and Hollan et al. (2000) describe, repair in human-human interaction is shared and grounded in mutual accountability. Robots, lacking social accountability and flexible interpretation, shift the burden of repair onto humans. One example of agency granted to Epi is seen in P8’s hesitation to intervene until Epi moves its hand away, suggesting respect for Epi’s physical agency. Responsibility is dynamically constructed and shifts with perceived competence. When Epi performs well, participants treat it as a responsible partner. When it fails, responsibility is reassigned, either to themselves (e.g., helping directly) or to the researcher (e.g., asking for help). This reflects a broader point: in HRI, responsibility is distributed and context-dependent. Failures trigger renegotiations of control, with gaze and speech revealing strategies for managing Epi’s limited communicative capacity.

### 6.2 What roles do participants take on during these interventions, and how does Epi’s role shift in the course of the interaction? How do agency and responsibility shift? (Q2)

As mentioned previously, a wide range of roles in HRI has been identified in the past (Onnasch and Roesler, 2021; Goodrich and Schultz, 2008; Yanco and Drury, 2004). While some of the roles participants took on corresponded to these existing taxonomies, in our analysis it became clear that roles shifted regularly, and that some of their nuances could not be captured within the technical taxonomies of collaborator, imitator etc. Building on feminist theories of research that emphasize situatedness and affect, our analysis was not only concerned with whether Epi was capable of performing the task. Rather, we also attended to the positions that participants were placed in when Epi failed and to the ways they navigated these situations. This perspective allowed us to recognize that participants’ responses were shaped not just by instrumental concerns, but also by relational, affective, and normative dynamics. Participants took on a range of roles in response to Epi’s errors; these roles were flexible, context-dependent, and shaped by participants’ interpretations of Epi’s competence and social character (Table 2). These roles often involved balancing task completion with the maintenance of a social script or relational dynamic. Some participants focused primarily on task efficiency (task-focused play), while others appeared more attuned to sustaining Epi’s role as a social partner. Below, we outline some of the roles participants adopted in response to Epi’s failures.

**TABLE 2:** Roles observed in moments of robot uncertainty and failure.

Role	Description
Participant as collaborator	Treated epi as a competent partner, working together on a shared task
Participant as mediator	Interprets the robot’s ambiguous actions to maintain interaction and agency, sometimes adjusting outcomes or redirecting communication
Participant as caregiver/nurturer	Gently assists the robot, mirrors its affect, and supports actions without taking over
Participant as moral agent/rule-follower	Follows turn-taking and fairness norms, smoothing over errors and preserving interaction integrity
Participant as observer/bystander	Steps back when others act, monitors cues, and stays ready to resume intervention
Researcher as facilitator	Guides participants, resets objects, and improvises solutions to support the robot
Researcher as takeover agent	Performs repairs or adjustments, causing participants to pause, withdraw, or defer responsibility

#### 6.2.1 Participant as collaborator

Certain participants, such as P8, treated Epi as a competent co-actor. They waited for Epi’s turn, interpreted its intended goals, and subtly fine-tuned the outcome without overriding its actions. This included coordinating actions, interpreting intent, and using embodied politeness, for example, avoiding physical overlap near Epi’s hand. This reflects a collaborative stance, reminiscent of how humans interact with novice agents like children or apprentices. It also resonates with the notion of scaffolding, where support respects the learner’s autonomy. P8’s subtle acknowledgment of Epi’s agency, waiting to adjust the ball only after Epi completed its action, demonstrates treating Epi as a co-present actor whose intentions and physical space mattered. The assistance was minimally invasive and preserved Epi’s agency. This contrasts with, for example, P2, whose interventions were sometimes more overriding when Epi was seen as less competent. In some cases, participants shifted from collaborator to proactive repair partner. For instance, P16 quietly repositioned balls nudged out of place without interrupting the flow. Epi then completed the turn successfully. This proactive repair was a subtle, anticipatory gesture correcting trajectory before full failure. Such support reduced future repair costs and actively scaffolded Epi’s capabilities. The participant was not merely reacting to breakdowns but facilitating smoother interaction by enabling the robot to succeed. This mirrors how a parent or tutor might gently support a learner without taking over. It reflects a non-intrusive helping role, preserving the robot’s agency while promoting success, and suggests a division of labor: the human allows space for Epi to self-repair, signaling trust in the robot and understanding of their supportive role.

#### 6.2.2 Participant as mediator

Some participants acted as mediators, interpreting Epi’s intent—especially during ambiguous or broken moments. They filled communicative gaps or ambiguities, helping sustain interaction and maintain Epi’s perceived agency. For example, P8 inferred where the ball was supposed to go and adjusted placement accordingly. In contrast, P2 saw similar ambiguity as a cue to escalate by appealing to the researcher. These mediation moments show how participants bridge the gap between robotic performance and human expectations, often maintaining the fiction of social competence. As Harrison and Johnson (2023) note, such ambiguity can be opportunities for relational engagement, sites where humans co-construct interaction, reconciling social robot imaginaries with practical system limits. A clear example is P12’s interaction, where the participant inferred intention despite Epi’s incomplete action, such as interpreting an empty-hand movement as meaningful (e.g., pointing to a target). This generous reading treated failed gestures as communicatively meaningful. Importantly, this interpretation required no additional action but upheld interaction coherence. The participant sustained Epi’s perceived intentionality and autonomy. This interpretive act reflects interactional generosity, compensating for execution failures by imputing plausible intention. Within Tian and Oviatt (2021)’s taxonomy, such moments correspond to failures in communicative function or social skill. Yet, participants like P12 repaired or reinterpreted robot behavior to preserve collaboration. This contrasts sharply with P2’s scene, where Epi’s failure ruptured agency recognition, and the participant shifted recipiency to the human experimenter, bypassing Epi. This redirection signals that P2 saw the failure as a breakdown in reciprocal social communication and prosocial interaction; Epi was no longer seen as a valid recipient of communication, and the participant’s behavior shifted accordingly.

#### 6.2.3 Participant as caregiver/nurturer

Some participants adopted a caregiving stance toward Epi, offering assistance that was gentle and affectively attuned. For example, P9 kept a respectful physical distance, rolled the ball delicately rather than grasping it outright, mirrored Epi’s affect (e.g., facial expressions), and leaned in affectively. Her movements seemed designed not to override Epi’s actions but to encourage and extend them. Such care was provided without overtaking the task; for instance, P9 did not pick up the ball for Epi but merely helped it roll in the right direction, treating Epi not as a malfunctioning tool but as a social subject. This behavior reveals an asymmetry of roles: the human becomes helper and caretaker, tending to the rhythm and tone of the interaction itself. However, the capacity to adopt such a caregiving role is not evenly distributed. Factors such as bodily ability, technical literacy, comfort with robotic systems, and cultural familiarity with technology shape whether individuals feel confident and safe enough to intervene (Halim et al., 2022; De Graaf et al., 2017; Rosenthal-von der Pütten et al., 2013; De Graaf and Allouch, 2013; Robertson, 2010; Nomura et al., 2008; Suchman, 2007). Hence, not everyone has the same interactional resources to take on this role, with implications for how HRI is designed and evaluated across diverse populations. In some cases, the caregiving role generated tension. For example, P14 helped Epi despite visible discomfort, displaying bodily hesitation, leaning back and forth, and avoiding contact with Epi’s hand. Her behavior reflected an internal negotiation between social norms to help and felt discomfort, what might be interpreted as a boundary violation or uncertainty about physical interaction with the robot. This underscores embodied social reasoning and mirrors observations by Denis (2019), who describe maintenance work as labor performed through and with the body. The discomfort could signal that Epi is not perceived as a mere object; hesitation to touch suggests social or moral weight, similar to touching a stranger. This caregiving role is also dynamic. As seen with P16, participants’ affective stance may shift over time. A less animated or more neutral response might reflect increasing familiarity, trust in Epi’s capabilities, reduced novelty, or emerging stable interactional expectations. Participants dynamically adjust their engagement and emotional orientation throughout the interaction.

#### 6.2.4 Participant as moral agent/rule-follower

Some participants oriented themselves to unspoken moral rules and general norms of maintaining order. For instance, P9 makes a face like they’ve done something naughty—such as intervening too much, and then smiles. This suggests internalized norms of fairness and turn-taking, like the idea that you do not pick up balls for others. Epi is thus treated not merely as a co-player but as a social subject embedded in a normative structure governing how to play properly. P16, among others, adjusts a ball even though it is no longer necessary for gameplay or the match is already over. These acts do not serve a pragmatic purpose but reflect a ritualized sense of contribution or a desire to preserve the rightness of the scene. Similarly, P12 acts as a maintainer of the social script, avoiding direct challenges or corrections to Epi’s moves and behaving as if the interaction is proceeding normally. This demonstrates alignment with social norms related to turn-taking and the game’s structure. As Denis et al. (2015) point out, maintenance and repair practices are situated within social worlds shaped by specific norms (Gregson et al., 2009), often giving rise to multiple, sometimes conflicting, forms of order. The robot’s errors are thus smoothed over, preserving the illusion of competence and continuity. Participants respect the game’s logic, for example, by not exploiting the robot’s mistake, and act as if Epi had completed its move, reflecting a concern for fair play and sustaining Epi’s legitimacy as a shared activity partner.

#### 6.2.5 Participant as observer/bystander

When the robot or researcher takes over the repair work, the human participant sometimes waits or defers, temporarily becoming more passive. This bystander role tends to be short-lived: when the researcher takes over a repair action or Epi initiates another attempt, participants often retreat slightly, observing rather than intervening. However, they typically remain alert and ready to step back in on the next turn if necessary. This behavior aligns with earlier described forms of monitoring and vigilance; participants momentarily withdraw because another agent is actively performing the task, but they remain attuned to the interaction and prepared to re-engage. It also involves active monitoring of contextual cues, such as noticing the researcher’s hand emerging from behind the curtain or subtle shifts in Epi’s behavior. This reflects an awareness of multiple agents in the scene and an ability to fluidly transition between active and passive modes of engagement.

#### 6.2.6 Researcher as facilitator

As described earlier, responsibility for repair in human-robot interaction is often distributed among co-present actors. In the case of Epi, the humans present, especially participants less familiar with the robot, may require guidance or reassurance about what actions are appropriate. This highlights how many technical artifacts, such as robots, extend beyond their physical form into a broader network of scaffolding and support that enables their effective functioning (Harrison and Johnson, 2023; Spektor et al., 2021). The researcher plays a crucial facilitative role, helping to bridge gaps in understanding and enabling smoother collaboration between human and robot. The researcher also facilitates the interaction by ‘inventing’ (cf. Denis et al., 2015) new ways of creating order, such as pushing the next ball to its starting position or resetting balls after failed attempts. To do so, the researcher often uses an extension of their hand: a custom-made ball pusher constructed from a ruler and a box. This shows how improvisation was not only required from the participant but also from the researcher to help the robot succeed.

#### 6.2.7 Researcher as a takeover agent

The presence of third-party scaffolding, where participants bypass the robot by interacting directly with the researcher, can be seen as the researcher taking over the interaction, thereby diminishing both Epi’s and the participant’s agency. For example, participant P8 initiates a helping attempt but withdraws their hand upon noticing the researcher’s hand emerging from behind the curtain to adjust the ball. In this context, the researcher’s earlier interventions might also shape participants’ expectations and may cue them to reassign responsibility, as observed with P2: When the researcher refrains from further intervention, the participant pauses and seeks clarification, highlighting how human involvement influences the perceived hierarchy of agency. This illustrates that human scaffolding can both undermine and reinforce robot agency within the interaction. It also matters whether participants see the researcher as controlling Epi, as this shapes how they perceive Epi and whether they consider certain actions necessary (e.g., if they already see Epi as an extension of the researcher, they might deem it less appropriate to interfere and help).

### 6.3 Factors facilitating or hindering repair and helping (Q3)

#### 6.3.1 Positionality of participants

Some participants appeared more comfortable stepping in to help or repair the interaction. Some were more playful or readily assumed a collaborative role with Epi. One key factor in understanding these differences is the positionality of the participants, researchers, and Epi itself. By positionality, we mean the intersection of social, cultural, and embodied factors, such as background, expertise, gender, and perceived authority, that influence how individuals interpret and act within the interaction (Secules et al., 2021). All participants were recruited from the vicinity of the university, which may have contributed to a sense of familiarity or confidence with experimental settings and technology. However, not everyone has the same opportunity or is granted the same legitimacy to improvise or assist a robot in ways that feel safe or appropriate. As described earlier, the ability and willingness to assist a robot are not evenly distributed. Factors such as bodily ability, technical literacy, prior familiarity with robotic systems, and comfort within specific sociocultural contexts all shape how participants engage with Epi. For instance, some participants physically stretched into Epi’s space to adjust or retrieve a ball, while others hesitated or used minimal gestures to avoid contact. These embodied differences reflect more than personal preference; they speak to how people experience their role in the interaction. Prior research supports this interpretation. Differences in comfort and engagement with robots have been linked to social background, prior exposure, attitudes toward robots, and broader cultural narratives about automation and assistance (Halim et al., 2022; De Graaf et al., 2017; Rosenthal-von der Pütten et al., 2013; De Graaf and Allouch, 2013; Robertson, 2010; Nomura et al., 2008; Suchman, 2007). Not everyone may have the capacity or resources to take on the role of helper, repairer, or collaborator in a robot-mediated task. Spatial arrangement and context: Another aspect in shaping participants’ helping and repair behavior was the spatial configuration of the interaction, particularly whether actions required reaching into Epi’s “territory” or physically interacting with the robot itself. Most participants were visibly hesitant to enter Epi’s action space, especially when doing so involved touching Epi or its hand. For example, if a ball landed too close to Epi, participants often paused, hesitated, or refrained entirely from intervening. This hesitation was likely influenced by an implicit boundary marking Epi’s workspace, which later also became the researcher’s as they stepped in to fix issues. The overlap between these zones made the space feel even less accessible to participants. Helping behaviors that required crossing this boundary often resulted in visible uncertainty. Participants (e.g., P14 and P8) leaned forward and then back again, stretched out their hands without completing the action, or glanced around before deferring to the researcher. The proximity and visibility of the researcher also seemed to play a role. Although seated behind a curtain, the researcher sat close to the table and occasionally reached in to adjust the setup. This proximity may have implicitly framed responsibility. In one case (P2), the participant initially helped Epi directly but later began to defer to the researcher during points of uncertainty. After seeing the researcher’s hand enter the frame during an earlier turn, the participant asked whether Epi needed help, no longer treating Epi as a fully intentional agent, but as something to be monitored by a human authority. When the researcher’s hand did not reappear, the participant seemed uncertain what to do.

#### 6.3.2 Boundary negotiation

Sometimes, Epi and the participant got physically too close, and boundaries had to be negotiated. For example, with P9, the participant’s hands almost got stuck with Epi’s, creating a moment of physical entanglement. However, the participant did not fully withdraw their hand or pick up the ball completely (which would have avoided Epi’s hand). This marks a fine-tuned negotiation of human–robot boundaries: when to help, how much, and with what affective tone. The participant lets Epi retain the lead role, even in a clumsy or compromised interaction. In addition to physical space, a key dimension emerges: the tension between social norms and physical comfort. It gives a glimpse of how humans internalize obligations toward robots as if they were moral agents, even amid ambivalence, ambiguity, or irrelevance to task success. For instance, P14 was visibly uncomfortable and unsure how close to get to Epi’s hand, but helped out anyway, perhaps not wanting to violate a social norm of assisting an agent in need. Yet it also shows participants’ bodily caution. Denis (2019) note that examining the distribution and nature of helping and repair labor is especially important due to its highly embodied and situated character. Some boundaries are not spatial but temporal and include respecting turn structure (e.g., waiting until Epi’s turn is over). Participants often allowed space for Epi to complete its action before intervening. Even when actions overlapped, they usually did so in ways that complemented Epi’s move, remaining part of its turn. Participants were generally careful not to interrupt, applying social norms to non-human agents and treating them as intentional beings with turns.

#### 6.3.3 Negotiations between participant and researcher

Another reason that repair was sometimes interrupted or hindered may have been the researcher’s taking-over of the task. This occasionally seemed unexpected from the participant’s perspective and could result in interrupted repair, e.g., in the cases of P2 and P14. At the same time, the triangular communication or scaffolding that occurred between the participant and the researcher might also have facilitated the repairing actions. Participants often seemed to receive confirmation about what they should do. This kind of triangular interaction resembles settings studied in developmental psychology, for instance, in experiments with parents and infants, where the parent looks at the baby but addresses the experimenter. Here, we see similar shifts of recipiency: the participant looks at Epi but talks to the researcher. There is a form of backchanneling, where the participant verbally addresses the researcher while the robot remains the (partial) recipient of the interaction. Such shifting of recipient roles, which is well documented in sociology (e.g., Goffman, 1981), shows how bystanders (like the researcher) are brought into conversations and help shape the trajectory of an action. In this context, the participant may be treating Epi as a side-recipient rather than a fully competent agent, addressing their concern to the researcher while still orienting bodily toward the robot.

### 6.4 Methodological reflections

This analysis benefited from combining different methods and types of data, each contributing specific insights into the dynamics of repair. Thick descriptions allowed us to observe aspects of the interaction that were difficult to capture within the constraints of the ELAN interface, such as moments of inaction, shifts in atmosphere, or contextual factors shaping participants’ behavior. These descriptions enabled a more holistic view of each situation and of the participants, including how they appeared to orient themselves emotionally and socially. The method was also more flexible, making it possible to include observations or associations that emerged during viewing, even if they were not part of a predefined coding scheme. By contrast, the multimodal conversation analysis (MCA) carried out using ELAN brought other advantages. It made the simultaneity of actions and the coordination of different modalities more visible and possible to track, facilitating a more detailed examination of how human and robot actions were intertwined. Both approaches focused on the human participant’s perspective, on what it made sense to do in the moment. At the same time, there were tensions between the methods. MCA avoids making claims about internal states or emotions, staying close to observable actions and their sequential organization (Mondada, 2018). Thick description, by contrast, allows for interpretive insights into affect, intention, and participant experience, helping clarify behaviors such as hesitation or embodied concern that might otherwise remain ambiguous. Together, the methods captured different dimensions of the interaction. For example, a participant’s hovering hand during an abandoned helping attempt might appear ambiguous in ELAN but gain clarity when contextualized through narrative description. We have already reflected on the positionality of participants as co-present actors in the scene, and how this relates to who can help and who feels ‘free’ to act. Another important aspect of positionality is the position of the robot used in this study. Epi is an active part of the knowledge production process. There is a difference between robots made by companies and those built in-house, which makes the knowledge production situated and dependent on context. We therefore have to point out that our observations are intimately tied to the specific robotic technology we used. Like everything else, Epi is an artifact created by specific people in the lab for specific purposes. In our case, it was created mainly for implementing the open-source cognitive modeling architecture Ikaros in a body and having a flexible robotic research platform. The robot therefore lacks features that commercial robots may have, due to fewer developers and limited troubleshooting resources. Commercial robots, such as Pepper, also have a ‘sealed-off’ body that hides, mystifies, and separates the technology from the user. In contrast, Epi is ‘open’ in several spots, making cables and motors visible, showing more of the ‘mess’ behind the smooth robot body. One useful concept to make sense of this is Norris’s (2004) notion of frozen action: objects or artifacts are shaped by prior social actions, which leave visible traces in later encounters. Some design choices were ad hoc solutions that did not anticipate real-world interaction and had to be adapted or repaired during interaction, for example, Epi’s hand needing to be at a very specific angle to succeed, or its twitching at the start of each new action sequence, which creates uncertainty about whether it has finished moving. Epi’s joints also make salient sounds when they move, undermining the illusion of effortless, humanlike motion by making effort audible. From a phenomenological perspective, interaction is a meaning-making process involving embodied coordination and mutual adjustment, often occurring without explicit planning or intention (Merleau-Ponty, 1962; Goodwin, 2000; Suchman, 2007). Participants’ limited understanding of Epi’s body and capabilities may make them cautious, since the robot might seem fragile, or unable to feel their touch, and thus pose a risk to them.

At the same time, our analyses are themselves situated. Following feminist STS, we acknowledge that what we recognized as actions of care or emotional displays is co-produced not only by participants’ actions and Epi’s material specificities, but also by our own positionalities as researchers. Our gendered socialization and disciplinary training may have oriented us to perceive and describe particular forms of responses to robot failure in accordance with our situated knowledges (Haraway, 2013).

### 6.5 Limitations and future directions

There are several limitations that should be acknowledged in this study. Although we draw on MCA in our analysis, a method often used in so-called in-the-wild studies, our data comes from a lab-based experiment with a relatively small sample, making it a less ecologically valid setting and limiting the generalisability of the findings. It is likely that humans would react differently to similar failures in more spontaneous environments (cf. Yamada et al. (2020); Mutlu and Forlizzi (2008)). The lab environment likely influenced participants’ behaviour: in real-world settings, people might disengage or walk away when a robot fails, whereas in the lab they were effectively required to remain and attempt to resolve the situation. The presence of researchers and the experimental framing may also have made the robot’s behaviour appear more intentional, blurring the line between natural interaction errors and experimental manipulations. Additionally, while the game of Tic-Tac-Toe allows us to examine failures within a turn-taking context, its predetermined structure limits the transferability of our observations to less structured settings. Several questions also remain open, such as how humans feel while performing the helping and repair behaviours described above. Since we did not collect interview data, we cannot make firm claims about felt experience of participants these interactions. Future studies could include explicit inquiries by using semi-structured interviews. Given our critical perspective on error and repair in HRI, collecting more personal information about participants would have allowed us to better consider issues of power and positionality. We suggest future research empirically test different categories of repair behaviour and examine how the researcher’s presence influences these behaviours (especially relevant in Wizard-of-Oz setups). Including interview data or participant narratives would help explore how participants perceive Epi’s agency (e.g., whether they see Epi as an extension of the researcher or as an independent actor), their own responsibility, and their feelings about caregiving and maintenance actions. Despite these constraints, this study offers a detailed categorisation of human roles and responses in moments of robot uncertainty and failure, including vigilance, mediation, caregiving, and moral rule-following. These insights can inform future research and design by highlighting how humans support robot functioning and manage social and moral responsibilities, potentially guiding the development of robots that safely leverage human assistance while protecting interactants from excessive burden.

## Data Availability

The raw data supporting the conclusions of this article will be made available by the authors, without undue reservation.

## References

[B1] Alač M. Movellan J. Tanaka F. (2011). When a robot is social: spatial arrangements and multimodal semiotic engagement in the practice of social robotics. Soc. Stud. Sci. 41, 893–926. 10.1177/0306312711420565 22400423

[B2] Albert S. De Ruiter J. P. (2018). Repair: the interface between interaction and cognition. Top. Cognitive Science 10, 279–313. 10.1111/tops.12339 29749039 PMC6849777

[B3] Anderson C. J. (2003). The psychology of doing nothing: forms of decision avoidance result from reason and emotion. Psychol. Bulletin 129, 139–167. 10.1037/0033-2909.129.1.139 12555797

[B4] Arnelid M. (2025). The imaginaries and politics of welfare technology: renegotiating elder care through technology for an ageing population. Linköping, Sweden: Linköping University Electronic Press (LiU Electronic Press). Ph.D. thesis.

[B5] Barad K. (2001). Re (con) figuring space, time, and matter. Feminist Locations Glob. Local, Theory Practice, 75109.

[B6] Blond L. (2019). Studying robots outside the lab: Hri as ethnography. Paladyn, J. Behav. Robotics 10, 117–127. 10.1515/pjbr-2019-0007

[B7] Brône G. Oben B. Jehoul A. Vranjes J. Feyaerts K. (2017). Eye gaze and viewpoint in multimodal interaction management. Cogn. Linguist. 28, 449–483. 10.1515/cog-2016-0119

[B8] Casilli A. A. (2025). “Waiting for robots: the hired hands of automation,” in Waiting for robots (University of Chicago Press).

[B9] Chevallier M. (2023). Staging Paro: the care of making robot (s) care. Soc. Stud. Sci. 53, 635–659. 10.1177/03063127221126148 36278323

[B10] De Graaf M. M. Allouch S. B. (2013). Exploring influencing variables for the acceptance of social robots. Robotics Autonomous Systems 61, 1476–1486. 10.1016/j.robot.2013.07.007

[B11] De Graaf M. Ben Allouch S. Van Dijk J. (2017). Why do they refuse to use my robot? Reasons for non-use derived from a long-term home study. Proc. 2017 ACM/IEEE International Conference Human-Robot Interaction, 224–233. 10.1145/2909824.3020236

[B12] de La Bellacasa M. P. (2011). Matters of care in technoscience: assembling neglected things. Soc. Studies Science 41, 85–106. 10.1177/0306312710380301 21553641

[B13] De Ruiter J. P. (2012). Questions: formal, functional and interactional perspectives, 12. Cambridge University Press.

[B14] Denis D. J. (2019). “Why do maintenance and repair matter?,” in The Routledge companion to actor-network theory (Routledge), 283–293.

[B15] Denis J. Mongili A. Pontille D. (2015). Maintenance and repair in science and technology studies. Tecnoscienza–Italian J. Sci. and Technol. Stud. 6, 5–15. 10.6092/issn.2038-3460/17251

[B16] Denzin N. K. (1989). Interpretive biography, 17. Los Angeles, London: SAGE Publications.

[B17] Dingemanse M. Enfield N. J. (2024). Interactive repair and the foundations of language. Trends Cognitive Sci. 28, 30–42. 10.1016/j.tics.2023.09.003 37852803

[B18] D’ignazio C. Klein L. F. (2023). Data feminism. MIT press.

[B19] Garza C. G. M. (2018). Failure is an option: how the severity of robot errors affects human-robot interaction. Pittsburgh: Carnegie Mellon University.

[B20] Giuliani M. Mirnig N. Stollnberger G. Stadler S. Buchner R. Tscheligi M. (2015). Systematic analysis of video data from different human–robot interaction studies: a categorization of social signals during error situations. Front. Psychology 6, 931. 10.3389/fpsyg.2015.00931 26217266 PMC4495306

[B21] Goffman E. (1981). Forms of talk. University of Pennsylvania Press.

[B22] Goffman E. (2023). “The presentation of self in everyday life,” in Social theory re-wired (Penguin Books Ltd.), 450–459.

[B23] Goodrich M. A. Schultz A. C. (2008). Human–robot interaction: a survey. Found. Trends® Human–Computer Interaction 1, 203–275. 10.1561/1100000005

[B24] Goodwin C. (2000). Action and embodiment within situated human interaction. J. Pragmatics 32, 1489–1522. 10.1016/s0378-2166(99)00096-x

[B25] Goodwin C. (2018). Co-operative action. Cambridge University Press.

[B26] Graham S. Thrift N. (2007). Out of order: understanding repair and maintenance. Theory, Culture and Society 24, 1–25. 10.1177/0263276407075954

[B27] Graziano V. Trogal K. (2019). Repair matters. Ephemera Theory Politics Organization 19, 203–227. 10.17613/NNYQ-7V91

[B115] Gregson N. Metcalfe A. Crewe L. (2009). Practices of object maintenance and repair: how consumers attend to consumer objects within the home. J. Consum. Cult. 9, 248–272.

[B28] Greiffenhagen C. Watson R. (2009). Visual repairables: analysing the work of repair in human–computer interaction. Vis. Commun. 8, 65–90. 10.1177/1470357208099148

[B29] Halim I. Saptari A. Perumal P. A. Abdullah Z. Abdullah S. Muhammad M. N. (2022). A review on usability and user experience of assistive social robots for older persons. Int. J. Integr. Eng. 14, 102–124. 10.30880/ijie.2022.14.06.010

[B30] Hamacher A. Bianchi-Berthouze N. Pipe A. G. Eder K. (2016). “Believing in bert: using expressive communication to enhance trust and counteract operational error in physical human-robot interaction,” in 2016 25th IEEE international symposium on robot and human interactive communication (RO-MAN) (IEEE), 493–500.

[B31] Haraway D. (1994). A manifesto for cyborgs: science, technology, and socialist feminism in. Postmodern Turn New Perspectives Social Theory 82.

[B32] Haraway D. (2013). “Situated knowledges: the science question in feminism and the privilege of partial perspective 1,” in Women, science, and technology (New York/London: Routledge), 455–472.

[B33] Harding S. (2009). Postcolonial and feminist philosophies of science and technology: convergences and dissonances. Postcolonial Stud. 12, 401–421. 10.1080/13688790903350658

[B34] Harrison K. Johnson E. (2023). Affective corners as a problematic for design interactions. ACM Trans. Human-Robot Interact. 12, 1–9. 10.1145/3596452

[B35] Harrison K. Perugia G. Correia F. Somasundaram K. van Waveren S. Paiva A. (2023). “The imperfectly relatable robot: an interdisciplinary workshop on the role of failure in hri,” in Companion of the 2023 ACM/IEEE international conference on human-robot interaction, 917–919.

[B36] Harrison K. Somasundaram K. Loutfi A. (2025). The imperfectly relatable robot: an interdisciplinary approach to failures in human–robot relations. What That Robot. Made Me Feel, 141–164. 10.7551/mitpress/15314.003.0009

[B37] Hayashi M. Raymond G. Sidnell J. (2013). Conversational repair and human understanding, 30. Cambridge University Press.

[B38] Heath C. Hindmarsh J. Luff P. (2010). Analysing video: developing preliminary observations. SAGE Visual Methods, 365–389. 10.4135/9781526435385.n4

[B39] Heritage J. (1984). Garfinkel and ethnomethodology. Cambridge: polity press.

[B40] Hoffman G. Cakmak M. Chao C. (2014). “Timing in human-robot interaction,” in Proceedings of the 2014 ACM/IEEE international conference on Human-robot interaction, 509–510.

[B41] Hollan J. Hutchins E. Kirsh D. (2000). Distributed cognition: toward a new foundation for human-computer interaction research. ACM Trans. Computer-Human Interact. (TOCHI) 7, 174–196. 10.1145/353485.353487

[B42] Honig S. Oron-Gilad T. (2018). Understanding and resolving failures in human-robot interaction: literature review and model development. Front. Psychology 9, 861. 10.3389/fpsyg.2018.00861 29962981 PMC6013580

[B43] Howes D. (2019). Multisensory anthropology. Annu. Rev. Anthropol. 48, 17–28. 10.1146/annurev-anthro-102218-011324

[B44] Iversen C. Persson M. Redmalm D. (2025). Playful framings of social robots in dementia care: reconsidering the principle of transparency in interactions with robot animals. Ageing and Soc. 45, 1585–1606. 10.1017/s0144686x24000539

[B45] Jackson S. J. (2014). 11 rethinking repair. Media technologies: essays on communication, materiality, and society, 221–239.

[B46] Jewitt C. (2015). “Multimodal analysis,” in The routledge handbook of language and digital communication (London, United Kingdom: Routledge), 69–84.

[B47] Johansson B. Tjøstheim T. A. Balkenius C. (2020). Epi: an open humanoid platform for developmental robotics. Int. J. Adv. Robotic Syst. 17, 1729881420911498. 10.1177/1729881420911498

[B48] Kendon A. (1967). Some functions of gaze-direction in social interaction. Acta Psychologica 26, 22–63. 10.1016/0001-6918(67)90005-4 6043092

[B49] Kim T. Hinds P. (2006). “Who should i blame? Effects of autonomy and transparency on attributions in human-robot interaction,” in ROMAN 2006-The 15th IEEE international symposium on robot and human interactive communication (IEEE), 80–85.

[B50] Kitzinger C. (2012). “Repair,” in The handbook of conversation analysis, 229–256.

[B51] Laprie J.-C. Arlat J. Beounes C. Kanoun K. (1995). “Definition and analysis of hardware-and-software fault-tolerant architectures,” in Predictably dependable computing systems (Springer), 103–122.

[B52] Latour B. Salk J. Woolgar S. (2013). Laboratory life: the construction of scientific facts

[B53] Lee J. D. See K. A. (2004). Trust in automation: designing for appropriate reliance. Hum. Factors 46, 50–80. 10.1518/hfes.46.1.50_30392 15151155

[B54] Lee M. K. Kiesler S. Forlizzi J. Srinivasa S. Rybski P. (2010). “Gracefully mitigating breakdowns in robotic services,” in 2010 5th ACM/IEEE international conference on human-robot interaction (HRI) (IEEE), 203–210.

[B55] Lefevor G. T. Fowers B. J. (2016). Traits, situational factors, and their interactions as explanations of helping behavior. Personality Individ. Differ. 92, 159–163. 10.1016/j.paid.2015.12.042

[B56] Lipp B. (2024). Robot drama: investigating frictions between vision and demonstration in care robotics. Sci. Technol. and Hum. Values 49, 318–343. 10.1177/01622439221120118

[B57] Luff P. Hindmarsh J. Heath C. (2000). Workplace studies: recovering work practice and informing system design. Cambridge, United Kingdom/New York: Cambridge University Press.

[B58] Mansoor K. M. A. (2025). The association between intolerance of uncertainty and psychological burden among caregivers of children with autism and the impact on their quality of life. Front. Psychiatry 16, 1492304. 10.3389/fpsyt.2025.1492304 40352369 PMC12061961

[B59] Martínez-Iñigo D. Totterdell P. Alcover C. M. Holman D. (2007). Emotional labour and emotional exhaustion: interpersonal and intrapersonal mechanisms. Work and Stress 21, 30–47. 10.1080/02678370701234274

[B60] Merleau-Ponty M. (1962). Phenomenology of perception. Humanities 67, 401–409.

[B61] Miller C. (2010). Character traits, social psychology, and impediments to helping behavior. J. Ethics and Soc. Phil. 5 (i), 1–37. 10.26556/jesp.v5i1.49

[B62] Mirnig N. Stollnberger G. Miksch M. Stadler S. Giuliani M. Tscheligi M. (2017). To err is robot: how humans assess and act toward an erroneous social robot. Front. Robotics AI 4, 21. 10.3389/frobt.2017.00021

[B63] Mondada L. (2018). Multiple temporalities of language and body in interaction: challenges for transcribing multimodality. Res. Language Social Interaction 51, 85–106. 10.1080/08351813.2018.1413878

[B64] Mukerji R. Merrill H. M. Erickson B. W. Parker J. Friedman R. (1991). Power plant maintenance scheduling: optimizing economics and reliability. IEEE Trans. Power Syst. 6, 476–483. 10.1109/59.76689

[B65] Mutlu B. Forlizzi J. (2008). “Robots in organizations: the role of workflow, social, and environmental factors in human-robot interaction,” in Proceedings of the 3rd ACM/IEEE international conference on human robot interaction, 287–294.

[B66] Nomura T. Kanda T. Suzuki T. Kato K. (2008). Prediction of human behavior in human–robot interaction using psychological scales for anxiety and negative attitudes toward robots. IEEE Transactions Robotics 24, 442–451. 10.1109/tro.2007.914004

[B67] Norris S. (2004). Analyzing multimodal interaction: a methodological framework. London, United Kingdom: Routledge.

[B68] Onnasch L. Roesler E. (2021). A taxonomy to structure and analyze human–robot interaction. Int. J. Soc. Robotics 13, 833–849. 10.1007/s12369-020-00666-5

[B69] Ostrowski A. K. Gunther L. DiPaola D. Breazeal C. (2025). “Power dynamics and autonomy: engaging employees around the design of autonomous agents,” in 2025 20th ACM/IEEE international conference on human-robot interaction (HRI) (IEEE), 909–918.

[B70] O’Hare G. M. Collier R. Ross R. (2004). “Demonstrating social error recovery with agentfactory,” in AAMAS’04 proceedings of the third international joint conference on autonomous agents and multiagent systems-volume 3 (IEEE).

[B71] Pelikan H. Hofstetter E. (2023). Managing delays in human-robot interaction. ACM Trans. Computer-Human Interact. 30, 1–42. 10.1145/3569890

[B72] Pelikan H. R. Reeves S. Cantarutti M. N. (2024). “Encountering autonomous robots on public streets,” in Proceedings of the 2024 ACM/IEEE international conference on human-robot interaction, 561–571.

[B73] Persson M. Iversen C. Redmalm D. (2025). “Robot animals and the emotional labor of caregivers,” in How that robot made me feel (MIT Press).

[B74] Ponterotto J. G. (2006). Brief note on the origins, evolution, and meaning of the qualitative research concept thick description. Qualitative Report 11, 538–549. 10.46743/2160-3715/2006.1666

[B75] Ragni M. Rudenko A. Kuhnert B. Arras K. O. (2016). “Errare humanum est: erroneous robots in human-robot interaction,” in 2016 25th IEEE international symposium on robot and human interactive communication (RO-MAN) (IEEE), 501–506.

[B76] Rawls A. W. Lynch M. (2024). Ethnography in ethnomethodology and conversation analysis: both, neither, or something else altogether? Qual. Res. 24, 116–144. 10.1177/14687941221138410

[B77] Reig S. Carter E. J. Fong T. Forlizzi J. Steinfeld A. (2021). “Flailing, hailing, prevailing: perceptions of multi-robot failure recovery strategies,” in Proceedings of the 2021 ACM/IEEE international conference on human-robot interaction, 158–167.

[B78] Rhee J. (2018). The robotic imaginary: the human and the price of dehumanized labor. Minneapolis, MN: University of Minnesota Press.

[B79] Robertson J. (2010). Gendering humanoid robots: robo-sexism in Japan. Body and Soc. 16, 1–36. 10.1177/1357034x10364767

[B80] Rogoff B. (2003). The cultural nature of human development. Oxford, United Kingdom: Oxford University Press.

[B81] Rosenthal-von der Pütten A. M. Krämer N. C. Hoffmann L. Sobieraj S. Eimler S. C. (2013). An experimental study on emotional reactions towards a robot. Int. J. Soc. Robotics 5, 17–34. 10.1007/s12369-012-0173-8

[B82] Sacks H. Schegloff E. A. Jefferson G. (1974). A simplest systematics for the organization of turn-taking for conversation. Language 50, 696–735. 10.2307/412243

[B83] Salem M. Lakatos G. Amirabdollahian F. Dautenhahn K. (2015). “Would you trust a (faulty) robot? Effects of error, task type and personality on human-robot cooperation and trust,” in Proceedings of the tenth annual ACM/IEEE international conference on human-robot interaction, 141–148.

[B84] Schieffelin B. B. Ochs E. (1986). Language socialization. Annu. Review Anthropology 15, 163–191. 10.1146/annurev.anthro.15.1.163

[B85] Schindler V. (2024). “Role assessments used in mental health,” in Assessments in occupational therapy mental health (Routledge), 321–339.

[B86] Schwennesen N. (2019). Algorithmic assemblages of care: imaginaries, epistemologies and repair work. Sociol. Health and Illness 41, 176–192. 10.1111/1467-9566.12900 31599986

[B87] Scollon R. Scollon S. W. (2003). Discourses in place: language in the material world. London: Routledge.

[B88] Secules S. McCall C. Mejia J. A. Beebe C. Masters A. S. L. Sánchez-Peña M. (2021). Positionality practices and dimensions of impact on equity research: a collaborative inquiry and call to the community. J. Eng. Educ. 110, 19–43. 10.1002/jee.20377

[B89] Sergi V. Hallin A. (2011). Thick performances, not just thick descriptions: the processual nature of doing qualitative research. Qual. Res. Organ. Manag. An Int. J. 6, 191–208. 10.1108/17465641111159152

[B90] Shapin S. (1989). The invisible technician. Am. Scientist 77, 554–563.

[B91] Silverman D. (1998). Qualitative research: meanings or practices? Inf. Systems Journal 8, 3–20. 10.1046/j.1365-2575.1998.00002.x

[B92] Spektor F. Rodriguez E. Shorey S. Fox S. (2021). “Discarded labor: countervisualities for representing ai integration in essential work,” in Proceedings of the 2021 ACM designing interactive systems conference, 406–419.

[B93] Star S. L. Strauss A. (1999). Layers of silence, arenas of voice: the ecology of visible and invisible work. Comput. Supported Cooperative Work (CSCW) 8, 9–30. 10.1023/a:1008651105359

[B94] Stedtler S. (2025). “Staging, accommodating or caring: reviewing the human labor involved in shaping robots into agents,” in 2025 20th ACM/IEEE international conference on human-robot interaction (HRI) (IEEE), 1650–1654.

[B493] Stedtler S. Fantasia V. Tjøstheim T. A. Brinck I. Johansson B. Balkenius C. (2025). Gaze and movement adaptation in response to delayed robotic movement during turn-taking. Sci. Rep. 15:34098. 10.1038/s41598-025-17140-9 41028111 PMC12485069

[B95] Stedtler S. Leventi M. (2025). “Who is responsible? Social identity, robot errors and blame attribution,” in Social robots with AI: prospects, risks, and responsible methods (Amsterdam: IOS Press), 284–297.

[B96] Steinbauer G. (2012). “A survey about faults of robots used in robocup,” in Robot soccer world cup (Springer), 344–355.

[B97] Suchman L. A. (2007). Human-machine reconfigurations: plans and situated actions. Cambridge: Cambridge University Press.

[B98] Suchman L. (2008). Feminist sts and the sciences of the artificial. Handbook Science Technology Studies 3, 139–164.

[B99] Tabatabaei R. Kostakos V. Johal W. (2025). “Gazing at failure: investigating human gaze in response to robot failure in collaborative tasks,” in 2025 20th ACM/IEEE international conference on human-robot interaction (HRI) (IEEE), 939–948.

[B100] Thunberg S. (2024). Companion robots for older adults: a mixed-methods approach to deployments in care homes, 878. Linköping, Sweden: Linköping University Electronic Press.

[B101] Tian L. Oviatt S. (2021). A taxonomy of social errors in human-robot interaction. ACM Trans. Human-Robot Interact. (THRI) 10, 1–32. 10.1145/3439720

[B102] Tomasello M. Carpenter M. (2007). Shared intentionality. Dev. Science 10, 121–125. 10.1111/j.1467-7687.2007.00573.x 17181709

[B103] Turkle S. (2011). *Life on the screen* (simon and schuster)

[B104] Van Der Woerdt S. Haselager P. (2019). When robots appear to have a mind: the human perception of machine agency and responsibility. New Ideas Psychol. 54, 93–100. 10.1016/j.newideapsych.2017.11.001

[B105] Veling L. McGinn C. (2021). Qualitative research in hri: a review and taxonomy. Int. J. Soc. Robotics 13, 1689–1709. 10.1007/s12369-020-00723-z

[B106] Verschuren P. J. (2001). Holism *versus* reductionism in modern social science research. Qual. Quantity 35, 389–405. 10.1023/a:1012242620544

[B107] Vinciarelli A. Valente F. Yella S. H. Sapru A. (2011). “Understanding social signals in multi-party conversations: automatic recognition of socio-emotional roles in the ami meeting corpus,” in 2011 IEEE international conference on systems, man, and cybernetics (IEEE), 374–379.

[B108] Wagner C. Wheeler L. (1969). Model, need, and cost effects in helping behavior. J. Personality Social Psychology 12, 111–116. 10.1037/h0027569 5784712

[B109] Winkle K. McMillan D. Arnelid M. Harrison K. Balaam M. Johnson E. (2023). “Feminist human-robot interaction: disentangling power, principles and practice for better, more ethical hri,” in Proceedings of the 2023 ACM/IEEE international conference on human-robot interaction, 72–82.

[B110] Winograd T. Flores F. (1986). Understanding computers and cognition: a new foundation for design, 335. Norwood, NJ: Ablex publishing corporation.

[B111] Yamada S. Kanda T. Tomita K. (2020). “An escalating model of children’s robot abuse,” in Proceedings of the 2020 acm/ieee international conference on human-robot interaction, 191–199.

[B112] Yanco H. A. Drury J. (2004). “Classifying human-robot interaction: an updated taxonomy,”2004 IEEE International Conference Systems, Man Cybernetics (IEEE Cat. No. 04CH37583). 3 2841–2846. IEEE, 10.1109/icsmc.2004.1400763

[B113] Zhou J. Arshad S. Z. Luo S. Chen F. (2017). “Effects of uncertainty and cognitive load on user trust in predictive decision making,” in Human-computer Interaction–INTERACT 2017: 16Th IFIP TC 13 international conference, mumbai, India, September 25-29, 2017, proceedings, part IV 16 (Springer), 23–39.

[B114] Žunjić A. (2018). Classification of causes of errors in the human-robot system. J. Robotics Mech. Eng. Res. 2, 7–13. 10.24218/jrmer.2018.26

